# Minimal Differences in Auditory and Visual Oddball Tasks in Autism: A Systematic Review and Meta-Analysis

**DOI:** 10.1007/s10803-025-06772-5

**Published:** 2025-03-08

**Authors:** Sarah G. Vassall, William J. Quackenbush, Mark T. Wallace

**Affiliations:** 1 Neuroscience Graduate Program, Vanderbilt University, Nashville, TN USA; 2 Vanderbilt Brain Institute, Vanderbilt University, Nashville, TN USA; 3 Department of Psychology, Vanderbilt University, Nashville, TN USA; 4 Department of Hearing and Speech, Vanderbilt University Medical Center, Nashville, TN USA; 5 Vanderbilt Vision Research Center, Nashville, TN USA; 6 Department of Psychiatry and Behavioral Sciences, Vanderbilt University Medical Center, Nashville, TN USA; 7 Department of Pharmacology, Vanderbilt University, Nashville, TN USA

**Keywords:** Sensory prediction, Oddball, Mismatch negativity, Auditory, Visual, Meta-analysis

## Abstract

Autism is a neurodevelopmental condition that presents with significant changes in sensory processing, and which has recently been associated with differences in sensory *expectations*. One method for measuring sensory expectations (i.e., predictions) is via oddball paradigms, in which a deviant stimulus is presented following a series of repeated stimuli. In EEG signals, this deviance elicits a characteristic mismatch negativity (MMN) response, which acts as a neural signature of deviance detection and perception. Given the growing focus on sensory prediction in autism, a number of studies have now employed the oddball paradigm, with mixed results. We conducted a meta-analysis to better understand the utility of oddball paradigms in evaluating sensory prediction differences in the autism population. A comprehensive literature search queried the PubMed database for empirical auditory and visual oddball studies comparing autistic and non-autistic individuals. Statistical analyses were all conducted in R. We estimated true effect sizes and characterized the effects of various study characteristics on effect size using a multi-level random effects model and robust variance estimation (RVE). Publication bias and study quality were also assessed. Although individual studies have reported differences, the results of this meta-analysis suggest no significant group differences between autistic and non-autistic individuals in auditory or visual oddball perception, recognition, or neural signatures. When used in autism research, auditory and visual oddball MMN responses may not inherently capture changes in sensory prediction, and significant findings may be related more to individual variability than diagnostic group.

**Supplementary Information:**

The online version contains supplementary material available at 10.1007/s10803-025-06772-5.

## Introduction

Autism is a neurodevelopmental condition that is characterized by social communication impairment (SCI) and patterns of restricted and repetitive behavior (RRB) (American Psychiatric Association, 2013). Additionally, sensory processing differences are considered part of the autism phenotype and have been shown to correlate with both SCI (Baranek et al., 2018; Foss-Feig et al., 2012; Lane et al., 2010) and RRB (Boyd et al., 2010; Foss-Feig et al., 2012; Schulz & Stevenson, 2019). The relationship between core autism features and sensory processing differences may lie in difficulty extracting and utilizing predictive sensory information from the environment.

Specifically, active engagement with the natural environment requires a degree of prediction of upcoming stimuli (e.g., via statistical regularities in the environment). For example, phonological and syntactic patterns in a given language restrict anticipated speech signals; use of complementary visual speech information, learned through experience, modulates auditory cortex activity (Callan et al., 2003; Calvert & Campbell, 2003; Möttönen et al., 2002; Sams et al., 1991; van Wassenhove et al., 2005) and facilitates comprehension and neural responses in both noisy (Ma et al., 2009; Plass et al., 2020; Rosenblum et al., 1996; Ross et al., 2007; Sumby & Pollack, 1954) and optimal (Crosse et al., 2015; Möttönen et al., 2002) environments.

Research on prediction in autism is long-standing and is proposed to be domain-general. Indeed, some relatively early neurological findings in autism include atypical brainstem and cerebellar structure and function (Courchesne & Allen, 1997; Courchesne et al., 1984; Grillon et al., 1989). For instance, visual and visuospatial tasks have revealed attentional orienting and shifting deficits in autism, which in many ways resembles performance by individuals with cerebellar damage (Courchesne & Allen, 1997; Courchesne et al., 1994; Townsend et al., 1996). Notably, the cerebellum integrates external sensory events, internal motor or cognitive preparations, and predictions of upcoming events. Atypical cerebellar function could then be expected to have widespread effects on formation and integration of predictions about the external environment. Contemporaneous with these neurological findings, the Complex Information Processing model (Minshew & Goldstein, 1998) was developed, which proposes that autism is characterized by intact performance on low-level motor and cognitive tasks, but deficits in more demanding, complex tasks (Minshew et al., 1997).

Combined, the historical perspectives point to a clear relevance between lower-order brain function and integration of environmental information and predictions. More recently, these theories have been expanded to be applied to the core features of autism. Most notably, RRBs such as rigidity in routines, and associated features—sensory sensitivities, difficulties with motion prediction, etc.—may be in part attributable to changes in sensory prediction abilities (see Sinha et al., 2014 for a review).

Yet it remains unclear if sensory prediction changes are widespread in the autism population. To evaluate the degree to which auditory and visual prediction differs between autistic and non-autistic individuals, we examined performance on auditory and visual oddball paradigms as a proxy for prediction. In oddball paradigms, a stimulus (e.g., a pure tone of a specific frequency) is presented repeatedly. After some pseudo-random number of repeats, a deviant stimulus (e.g., a pure tone at a different frequency) is presented. This paradigm can be used to elicit neural signatures of pattern violation and change detection. Despite the ubiquity of the oddball paradigm and its easy application to autism, research in this area is fraught with inconsistencies, no doubt partly due to the heterogeneity of the autism population and innumerable possible variations in the oddball paradigm (i.e., the stimulus itself). However, if the oddball paradigm is a strong measure of prediction, and prediction is altered in autism, then one would expect a meta-analysis to provide support for these associations.

### Examining Sensory Processing in Autism from a Predictive Coding Perspective

Building off of historical theories of altered prediction abilities, computational research in autism suggests that complex core and associated features of autism may be driven by differences in sensory expectations (Pellicano & Burr, 2012; Zaidel et al., 2015; Van de Cruys et al., 2021; Noel et al., 2020; Park et al., 2017; Amoruso et al., 2019; Chambon et al., 2017). Predictive coding refers to the idea that the brain, faced with an uncertain environment, represents stimuli according to a probabilistic distribution. That is, top-down expectations about the environment are constantly updated based on incoming bottom-up sensory information; simultaneously, perception of bottom-up sensory stimuli is shaped by those top-down expectations. The predictive coding framework assumes that the goal of prediction is to minimize prediction errors (see Friston, 2009; Tarasi et al., 2022 for comprehensive reviews).

However, in autism, it can be difficult to disentangle the bottom-up and top-down components when both domains may well be altered. The true answer is likely to be nuanced and to encompass some degree of both bottom-up and top-down irregularities. For instance, habituation—characterized by the reduction in neural responses to a repeated stimulus—is largely found to be altered in autism. Reduced habituation of auditory N1 (Hudac et al., 2018), P1 (Cary et al., 2024; Ruiz-Martínez et al., 2020), and N2 (Dwyer et al., 2023) components combined with prolonged looking times to repeated visual stimuli (Hocking et al., 2023; Webb et al., 2010)—suggest a modality-general difference in habituation in autism. However, from these findings it is unclear if autistic individuals are slow to habituate due to atypical sensory processing or reduced reliance on prior stimulus exposure to inform predictions.

Indeed, changes in sensory prediction may be related to autistic individuals’ differences in their ability to contextualize (Palmer et al., 2017; Pellicano & Burr, 2012) or flexibly incorporate (Van de Cruys et al., 2014) new sensory information (i.e., top-down). Autistic individuals frequently exhibit enhanced low-level sensory discrimination abilities (e.g., for luminance-defined sinusoidal gratings (Rivest et al., 2013) and auditory tones (Bonnel et al., 2010; Heaton, 2003; Heaton et al., 2008)), but decreased discrimination ability for higher-order stimuli (e.g., prosody; Brooks et al., 2018; Korpilahti et al., 2007, see Samson et al., 2006 for a review). Combined, this evidence simultaneously points to enhanced perceptual acuity for simple, low-level stimulus features, but perceptual deficits that increase with stimulus complexity. Furthermore, autistic individuals with otherwise average cognitive profiles exhibit changes in performance on complex—but not simple—motor, language, and memory tasks (Minshew et al., 1997). This finding may point to domain-general difficulties integrating higher-order information.

When considering the role of sensory prediction in the presentation of autism features, it is reasonable to believe that prediction difficulties extend to—or indeed are amplified in—social contexts: ability to contextualize individuals’ actions and predict subsequent actions and mental states likely builds substantially on lower-level sensory prediction ability. Importantly, studies of social prediction in autism have revealed decreased anticipatory eye movements (Arthur et al., 2023) and use of communicative gestures (von der Lühe et al., 2016) to inform action perception.

### The Present Study—A Meta-Analysis of the Oddball Paradigm

The purpose of this study was to conduct a meta-analysis examining group differences across auditory and visual oddball paradigms in autism—a paradigm specifically used to elicit neural signatures of pattern violation and change detection. Although other neural components were included in some of the papers cited in this meta-analysis (e.g., global field potential), our primary neural metrics of interest—and indeed those which are most widely associated with event-related potentials (ERPs) to deviant stimuli—were the N1, N2, and P3 components. We were also interested in whether other paradigm and sample characteristics might modulate effect size.

For instance, we hypothesized that effect sizes would be larger for more complex stimuli. Not only are sensory processing differences in autism more pronounced for increasingly complex stimuli, as discussed above, but also, studies of N1 have shown it to be modulated by stimulus features (Rugg & Coles, 1995) and task type (Vogel & Luck, 2000). Similarly, P3 is hypothesized to function as a part of context updating, wherein a deviant stimulus causes an individual to update their expectations about the sensory environment (Polich, 2007). Thus, we would expect N1 and P3 effect sizes to be modulated by stimulus properties and complexity.

Additionally, event-related potential (ERP) amplitudes and latency show substantial change over the course of development (e.g., N1, see (Tomé et al., 2015) for a systematic review and meta-analysis; N2, see (Morr et al., 2002; Tomé et al., 2015); P3, (see (van Dinteren et al., 2014) for a systematic review and meta-analysis). We therefore hypothesized that participant age would be a modulating factor in ERP effect sizes. We also anticipated that degree of autism presentation would be related to effect sizes. N2, which comprises mismatch negativity (MMN) and N2b, encompasses *pre-conscious* detection of stimulus change (Ritter et al., 1979) and attentional allocation (i.e., *conscious* awareness) toward stimulus changes (Näätänen et al., 2007), respectively. Attentional differences are quite common in autism, and we expected that degree of functional challenges might correlate with the degree of group differences observed in the neural signals.

Finally, given the increased rate of autism diagnosis in males compared to females, we sought to account for sex in our analyses. Few studies have examined sex effects on oddball ERPs in healthy populations—and none that we are aware of in autism—although it appears that, largely, there are no sex differences (e.g., see Polich et al., 1985; Criel et al., 2023; Kamp et al., 2021) for studies of sex differences in P3 latencies). Thus, we expected no effect of sex on effect size.

## Methods

### Search Strategy & Inclusion/Exclusion Criteria

For each modality—auditory and visual—a comprehensive literature search queried the PubMed database. For the auditory modality, search terms included ((auditory adaptation) OR (auditory mismatch negativity) OR (auditory oddball)) AND (autism); for the visual modality, search terms included ((visual adaptation) OR (visual mismatch negativity) OR (visual oddball)) AND (autism). For all included studies (auditory *N* = 42; visual *N* = 15), manual searches of the reference section (reverse reference search) were also conducted to retrieve relevant studies not identified in the initial search (auditory *N* = 18; visual *N* = 0). Additionally, for each study selected from the initial PubMed query, we identified additional relevant studies that cited those papers (forward reference search) (auditory *N* = 4), with the final search updated on January 2, 2024. Studies were included in the final analysis if they met all the following criteria:

- Reports neurophysiological methods (EEG, fMRI, etc.) with sufficient information to calculate a standard difference effect size
- Participant samples include autistic and non-autistic controls or infants at high and low familial risk for autism
- Available in English

Studies were excluded from analysis if they met any of the following criteria:

- No idiopathic autism/high-risk group (syndromic autism such as Fragile X Syndrome excluded from analyses)
- No healthy control group
- No neurophysiological or behavioral method relevant to the search criteria
- Animal models
- Therapeutic intervention
- Non-primary literature (i.e., meta-analyses, reviews, commentaries)
- Duplicates
- Unable to be retrieved

A content expert examined each title and abstract for inclusion. Full text reviews were conducted for studies that met initial title and abstract inclusion.

### Study Coding

The first author extracted and coded each study. Study characteristics—study type (diagnosed, at-risk), mean age, percent male, autism severity scores and diagnostic system used—were collected, as well as the type of oddball discriminator and stimulus used and the variable of interest. When available, means and standard deviations or standard errors were recorded for each participant group; if reported by the author in lieu of the mean, the t-statistic, F-value, t-test p-value, or chi-square values were used to calculate effect size.

Effect size (Cohen’s *d*) was calculated for each data point using a web-based effect size calculator (Wilson, 2023). To ensure rater reliability, all effect sizes were validated by the second author or additional lab members (acknowledged) who were blinded to all study hypotheses. Reliability was excellent (absolute agreement = 94.7%). Disagreements were due to human error during initial data entry.

It should be noted here that the studies analyzed rarely discriminated between the N2 components MMN and N2b, instead referring to them both interchangeably as the N2 component. For analytical completeness, the authors simply coded these variables as N2.

### Statistical Analyses

All statistical analyses were conducted in R (R Core Team, 2017). We used the *metafor* (Viechtbauer, 2010) package’s standard formulas to convert Cohen’s *d* to Hedges’ *g*. We chose Hedges’ *g* for its ubiquity and interpretability, as well as consideration of sample size in its calculation. Additionally, both Cohen’s *d* and Hedges’ *g* easily can be calculated from a variety of reported statistics (e.g., F-values). All analyses described hereafter were conducted independently on auditory EEG/imaging data, auditory behavioral data, visual EEG/imaging data, and visual behavioral data.

We first examined average effect sizes for each study. Using the *metagen* function in the *meta* (Balduzzi et al., 2019) package, we created a random-effects model, which assumes variable weights for each study. We first sought to estimate true effect size differences by calculating between-study heterogeneity. For each study, we calculated the estimated standard deviation of the true effect size (τ), the estimated between-study heterogeneity (τ^2^), and the prediction interval of the true Hedges’ *g*. To identify which datapoints most contributed to between-study heterogeneity, we used the *find.outliers* function in *dmetar* (Harrer et al., 2019) which searches for statistical outliers in a fixed- or random-effects model. In this package, outliers are defined as data for which the upper bound of CI 95% < the lower bound of the prediction interval or for which the lower bound of the CI 95% > the upper bound of the prediction interval (Harrer et al., 2019). While these studies were not excluded from further analysis, we were able to observe the effect of outlier removal on between-study heterogeneity.

We next explored publication bias by conducting an Egger’s regression test (Egger et al., 1997) via the *metabias* function of *meta* (Balduzzi et al., 2019). For our analyses, we calculated a linear regression of the Hedges’ *g* values—weighted by their inverse variances—on their standard errors. We then conducted a 1-sample t-test to determine whether the estimated bias was significantly larger than 0. Note that we were unable to complete this step for the auditory behavioral studies, as there were too few to fit a robust regression model (*N* = 9).

Using individual data points, we next sought to characterize the influences of various study characteristics effect size. To do so, we first selected a model. For each dataset, we generated 2 multilevel random-effects models using the *mra.mv* function in *metafor* (Viechtbauer, 2010): a 2-level (study-level, pooled; Eq. 1) model and a 3-level (effect size-level, study-level, pooled; Eq. 2) model. We then compared performance by calculating a standard analysis of variance (ANOVA). If one model significantly outperformed the other, that model was selected for further analyses; if model performance was not significantly different, we selected the more parsimonious 2-level model.

Upon selecting the most appropriate model, we aimed to assess variables that may moderate the pooled effect size using the following model structure:

1 $$hedgesg \sim moderator + re_{study}$$

2 $$hedgesg \sim moderator + re_{study} + re_{effectsize}$$

For all datasets, moderators of interest included study type (diagnosed, at-risk), oddball discriminator (e.g., frequency), stimulus type (e.g., tone), task (active or passive), stimulus emotionality, mean age, and percent male. For EEG/imaging studies, we also included the component (e.g., N2 amplitude) as a moderator, both alone (see Eqs. 1–2) and, if significant, in conjunction with the stimulus (oddball discriminator and stimulus type) and task (i.e., active vs. passive) parameters (Eq. 3):

3 $$hedgesg \sim component*oddball discriminator*stimulus*activity + re_{study}$$

For behavioral studies, we included both the type of behavioral task (e.g., detection) and the behavioral metric (e.g., response time).

Although not a part of our a priori hypotheses about effect size differences, it was noted that the first auditory oddball study cited was published in 1984, whereas the first visual auditory study cited was published in 2009 (see Table 1 below). Thus, we included publication year as an additional moderator to explore how technology and perspectives on sensory prediction may have influenced effect size. Finally, to elucidate any connection between social communication ability and sensory prediction, we compared effect sizes for emotional or affective stimuli (e.g., for tasks requiring emotion discrimination) with those of neutral stimuli. For each model, we evaluated the p-value of each coefficient to identify significant predictors. Additionally, for categorical moderators, we conducted an F-test of moderators.

**Table 1:** Search results

*Auditory (N* = *64)*			
Publication dates	1984–2024		
Diagnosed	98%		
Participant characteristics	Autism mean (± SD)	Non-autism mean (± SD)	p
Age	13.5 (± 8.7)	14.2 (± 8.9)	0.68
% Male	80.8 (± 12.2)	69.9 (± 17.5)	0.002*
Mean effect size	0.16 (± 2.42); range [-5.33, 33.44]		

*Visual (N* = *15)*			
Publication dates	2009–2022		
Diagnosed	84%		
Participant characteristics	Autism mean (± SD)	Non-autism mean (± SD)	p
Age	18.5 (± 11.2)	18.6 (± 10.6)	0.98
% Male	83 (± 9.2)	75.5 (± 11.8)	0.14
Mean effect size	0.27 (± 1.93); range [-6.76, 10.48)		

^*p < 0.05^

**Fig. 1:**
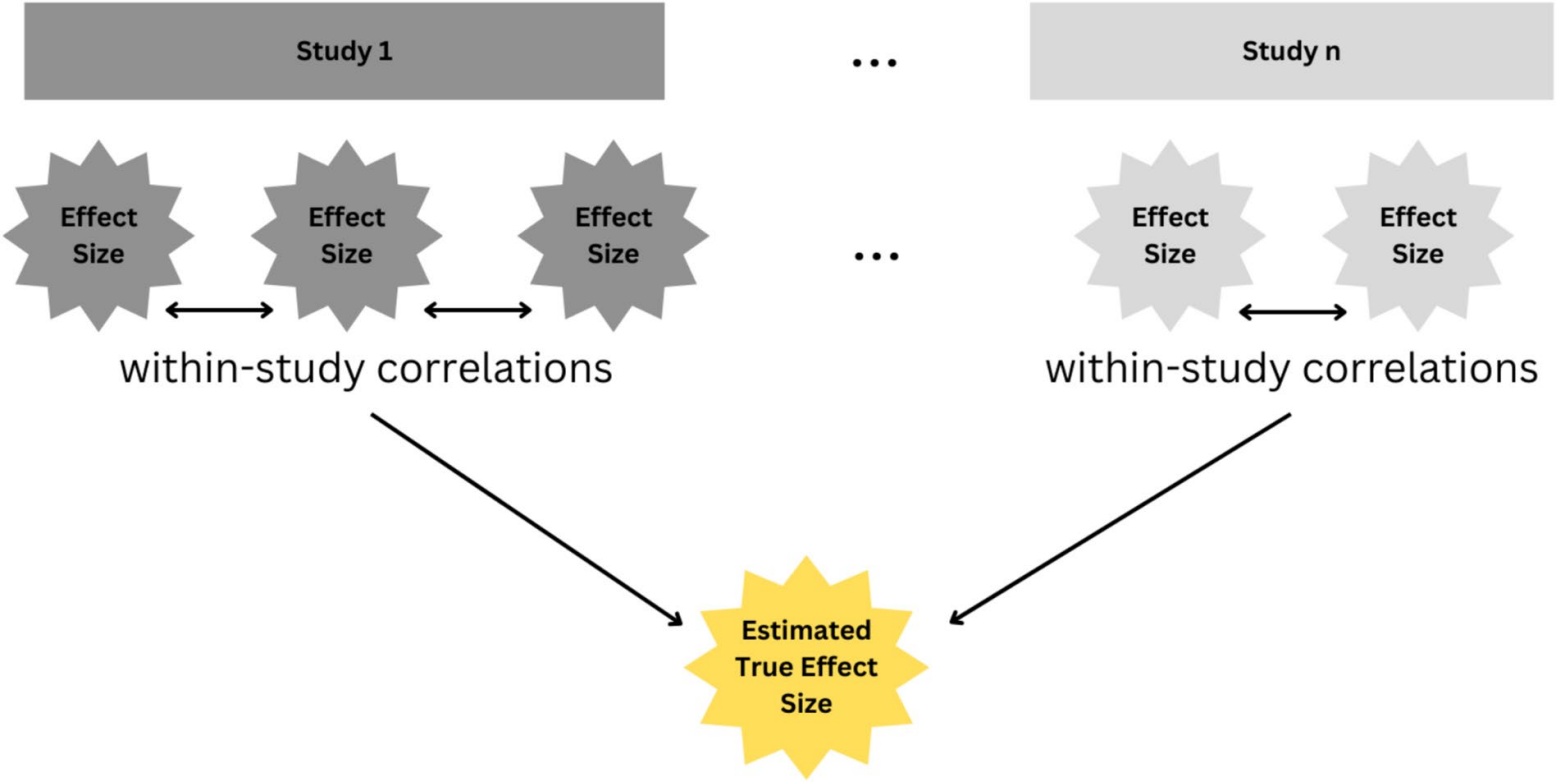
Schematic illustrating the rationale for robust variance estimation (RVE). RVE, unlike standard models used to evaluate effect size differences, assumes inter-correlation between the effect sizes within a given study

Finally, for any model results with significant effects, we repeated multilevel analyses using robust variance estimation (RVE) to verify the significance of the result. Unlike standard multi-level models—which do assume dependence between effect sizes nested within a level, but also assume that effect size estimates are independent between studies (Hedges et al., 2010)—RVE does not make this independence assumption. We a conducted hierarchical RVE using the *clubSandwich* (Pustejovsky, 2022) package, which assumes a priori that effect sizes from a single study are correlated, as the same subjects’ data within a given study are being used to generate a multiple effect sizes (Fig. 1). When building an RVE, we had to pre-define a correlation coefficient, ρ, between effect sizes within the same study. To begin, we assumed a large correlation, assigning ρ = 0.6. We next conducted a sensitivity analysis using the *sensitivity* function in the package *robumeta* (Fisher & Tipton, 2015) and a hierarchical weighting scheme, which allowed us to evaluate whether our estimate was sufficiently robust across different ρ values ranging from 0 to 1.

### Study Quality

In accordance with Preferred Reporting Items for Systematic Reviews and Meta-Analyses (PRISMA) guidelines, we coded each study’s overall quality. Study quality metrics were adapted for autism from the Newcastle–Ottawa Scale (NOS) (Wells et al., 2014) as used by other meta-analyses of similar areas of focus (Williams et al., 2023). The key metrics of interest are outlined in Supplementary Table 1. Each study was manually coded for quality by the first author and verified by the second author. Reliability was excellent (absolute agreement = 97.8%).

## Results

### Search Results

**Fig. 2:**
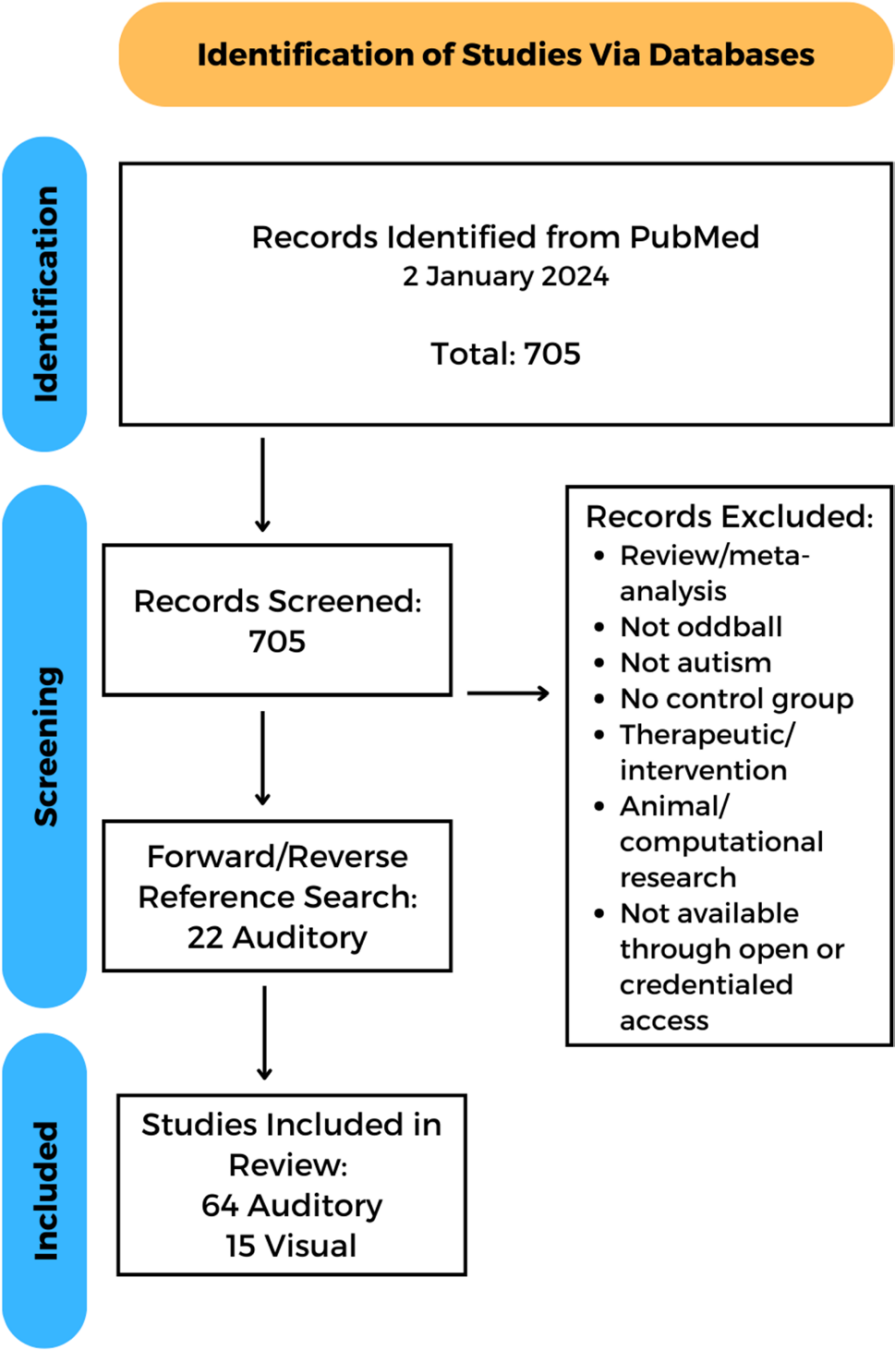
PRISMA diagram illustrating publication search and screening process

The initial PubMed query for auditory studies yielded 209 results, with 42 studies being retained. Forward and reverse citation searches yielded another 22 studies, for a total of 64 studies, including 496 imaging effect sizes and 48 behavioral effect sizes. Nine of these studies included behavioral data. For the visual search, the initial query generated 448 results, and no additional articles were found through forward or reverse citation searches. A total of 15 studies were retained, including 74 imaging effect sizes and 42 behavioral effect sizes. Ten of these studies included behavioral data (Fig. 2).

Studies used for this meta-analysis are found in Supplementary Table 2. Although the majority of auditory studies used EEG, some of the studies cited used MEG (*N* = 7) and fMRI (*N* = 2); the remaining 55 studies used EEG to characterize brain responses to auditory oddballs; all visual studies used EEG. Study characteristics are displayed in Table 1.

### Effect Size Heterogeneity

#### Auditory Oddball

**Fig. 3:**
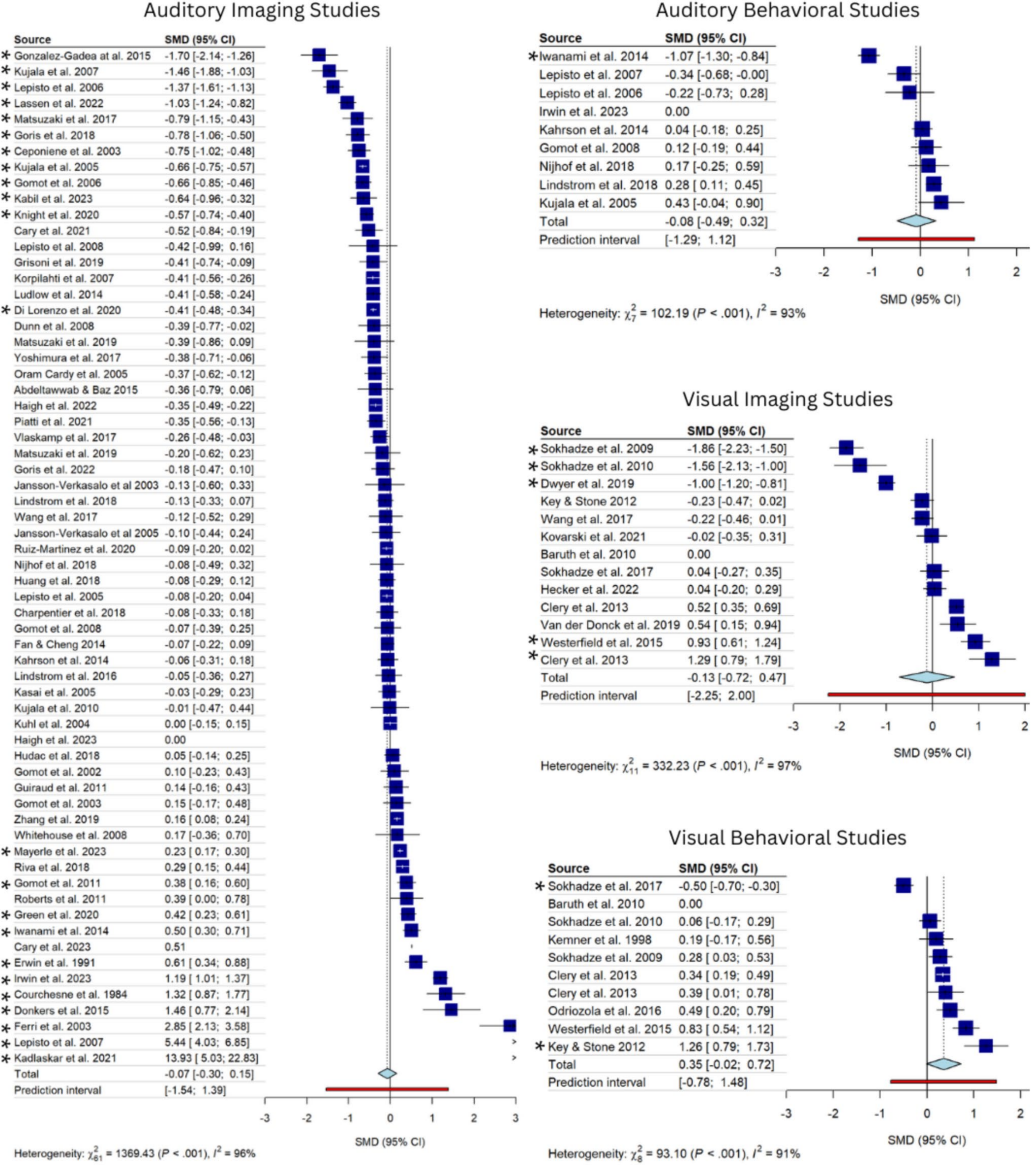
Effect sizes of **a** auditory imaging studies, **b** auditory studies with additional behavioral data, **c** visual imaging studies, and **d** visual studies with additional behavioral data. Blue diamonds indicate pooled estimate and variance. Studies identified as potential outliers marked with *

The estimated true effect size of all studies combined (Fig. 3a) was *g* = − 0.07, with a 95% CI of [ − 0.3, 0.15]. Between-study heterogeneity was high (τ^2^ = 0.52); the estimated standard deviation of the true effect sizes was τ = 0.73, with the majority of variation driven by true differences in effect size (I^2^ = 95.5%). However, it is important to note that the range of effect sizes was large, and we anticipated that outliers were the primary driver of these differences. Using the *find.outliers* function, we identified 24 potential outlier studies; when excluded, the estimated true effect size was somewhat larger with a narrower prediction interval (*g* =  − 0.13; [− 0.2; − 0.06]). Excluding these outlying studies also yielded a much smaller between-study heterogeneity score and effect size variance (τ^2^ = 0.03, τ = 0.17), although true differences in effect size still accounted for the majority of study variation (I^2^ = 75.8%). However, it should be noted that the majority of the studies identified as outliers used speech stimuli, which are likely to generate highly heterogeneous results simply as a function of linguistic ability and overall level of support received by individuals in the autistic sample. Thus, we did not eliminate these studies from further analysis, but rather accounted for sample characteristics in our analyses below.

When comparing only studies that contained additional behavioral data (Fig. 3b), the estimated true effect size was *g* =  − 0.08, with a 95% CI of [− 0.49, 0.32]. We observed a fairly small between-study heterogeneity score (τ^2^ = 0.21); the estimated standard deviation of the true behavioral effect sizes was τ = 0.46, with the majority of the variation in the data estimated to come from true differences in effect size (I^2^ = 93.1%). The test of heterogeneity was significant (Q = 102.19; p < 0.0001). We identified 1 potential outlier.

From these analyses, we can conclude that there are minimal overall neural or behavioral group differences in auditory oddball paradigms, as evidenced by the small estimated true effect sizes, with CIs that include 0. Furthermore, there is significant between-study heterogeneity in effect sizes, which makes it difficult to interpret auditory oddball results in the context of prior literature.

#### Visual Oddball

The estimated true effect size of the visual imaging dataset (Fig. 3c) was *g* =  − 0.13, with a 95% CI of [− 0.72, 0.47]. Heterogeneity between studies was even larger in the visual imaging dataset than in the auditory, with a heterogeneity score of τ^2^ = 0.84 and an estimated standard deviation of the true effect size of τ = 0.92. Almost all effect size variance was estimated to be true differences (I^2^ = 96.7, Q = 332.23, p < 0.0001). Five studies were identified as potential outliers. When removed, the estimated true effect size is *g* = 0.09 with a 95% CI of [− 0.2, 0.39], an estimated standard deviation of τ = 0.29, and a between-study heterogeneity score of τ^2^ = 0.08 (Q = 42.43, p < 0.0001). Even when the potential outlying studies are removed, the majority of variance is estimated to be from true differences in effect size (I^2^ = 85.9%).

Comparisons of only studies with behavioral data (Fig. 3d) revealed an estimated true effect size of *g* = 0.35, with a 95% CI of [− 0.02, 0.72]. Relative to the auditory behavioral estimate, we observed a similar between-study heterogeneity score (τ^2^ = 0.2) and estimated standard deviation of the true behavioral effect size (τ = 0.45). The majority of the effect size variance was estimated to stem from true differences in effect size (I^2^ = 91.4%). The test of heterogeneity was also significant (Q = 93.1, p < 0.0001). We identified 2 potential outlier studies using the *find.outliers* function, which when removed, yielded a much smaller—though still significant—study-level heterogeneity (Q = 18.82, p = 0.005).

As with the auditory studies, we observed minimal overall neural and behavioral group differences for visual oddball paradigms: both estimated pooled effect sizes were very small, with CIs that included 0. Additionally, between-study heterogeneity was even higher in the visual studies, indicating a lack of generalized findings against which to compare study results.

### Publication Bias

**Fig. 4:**
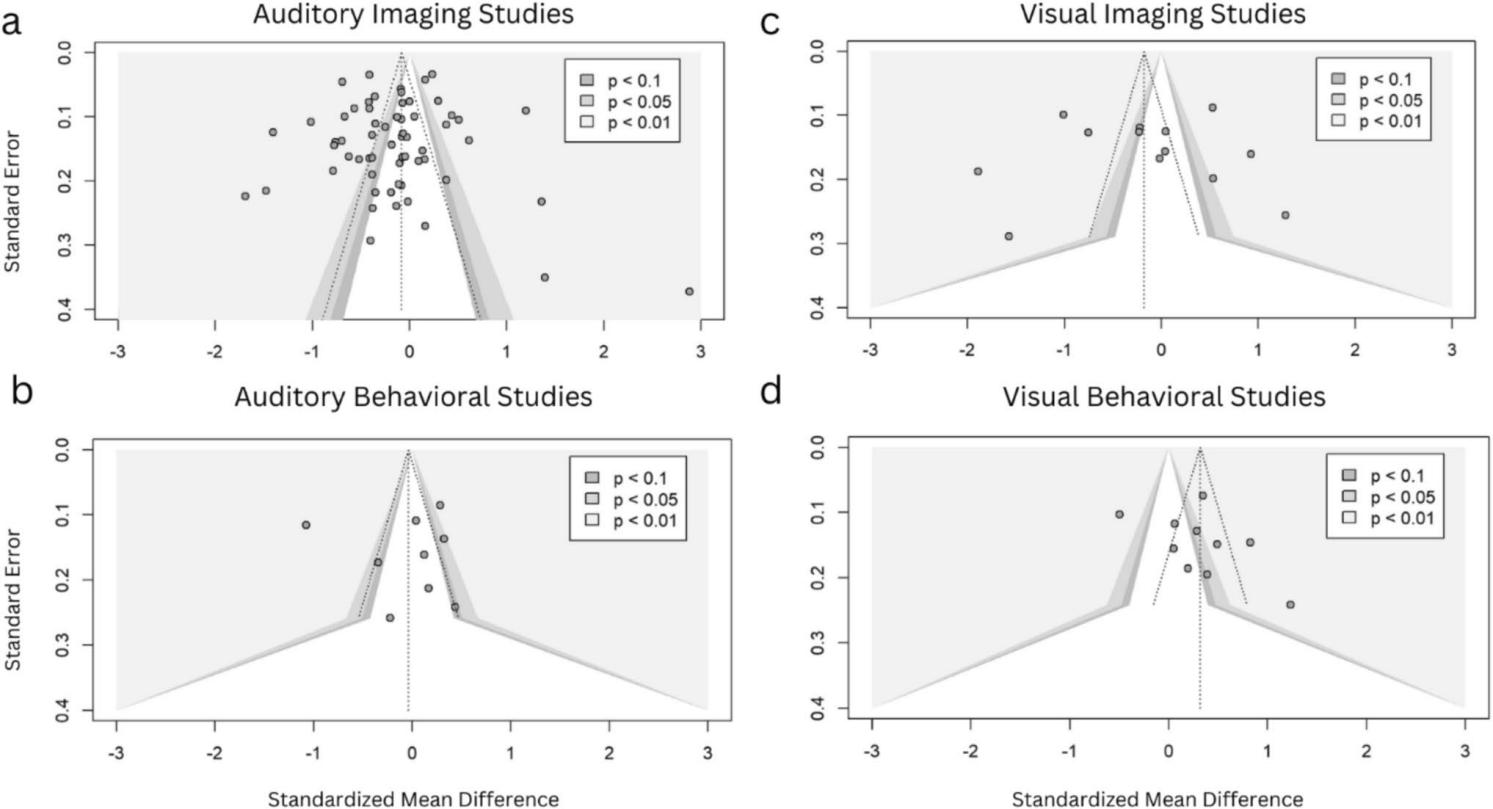
Publication bias for **a** auditory imaging studies (outliers removed from visualization), **b** auditory behavioral studies, **c** visual imaging studies, and **d** visual behavioral studies. Egger’s regression test could not be completed for auditory behavioral studies due to small sample size. The *x*-axis represents average study effect size; the *y*-axis represents average study standard error

The purpose of examining publication bias is to better understand to what degree studies with non-significant findings may be withheld publication. Publication bias may pose a significant problem for researchers aiming to understand generalized findings. We used Egger’s regression test to calculate the regression intercept of scaled study effect sizes regressed against their standard errors. Funnel plots may also be used to visualize publication asymmetry—an indirect examination of publication bias that indicates the presence of studies with exceedingly positive or negative effect sizes. As shown in Fig. 4, the vertical line represents the estimated pooled effect, and each study is plotted by its average effect size (*x*-axis) and standard error (*y*-axis). Studies should fall within a funnel shape, wherein a wide funnel distribution indicates a great deal of study heterogeneity. Additionally, the color contours shown in Fig. 4 show the average significance level of each study.

#### Auditory Oddball

For the sake of visualization, outliers have been removed from the funnel plot of auditory imaging studies (Fig. 4a), but all studies were included in the Egger’s regression test. Despite the large variation in effect sizes and high number of identified outliers, study asymmetry was not significantly larger than 0 (t = 0.23, p = 0.8182). Nonetheless, these results should be taken with caution: Fig. 4aclearly demonstrates a number of studies with unusually large negative and positive effect sizes relative to the pooled estimate. The resulting non-significant asymmetry calculation may be a result of the relatively low effect size standard errors across many of the studies.

Our estimation of publication bias across the auditory behavioral studies could not be completed due to the small sample size (*N* = 9). However, visual inspection of the corresponding funnel plot (Fig. 4b) shows that all studies fall within or very nearly within the ideal funnel, suggesting minimal asymmetry in publication.

#### Visual Oddball

Publication bias was not significant for either visual imaging studies (t =  − 0.12, p = 0.9053; Fig. 4c) or visual behavioral studies (t = 1.03, p = 0.3315; Fig. 4c). However, as with the auditory imaging studies, Fig. 4c displays a number of unusually highly positive and negative effect sizes in the imaging studies of visual oddballs.

### Multilevel Modeling and Robust Variance Estimation (RVE)

As described in Sect. 2, each dataset was first compared using a 2-level (study-level, pooled) mixed effects model and a 3-level (effect size-level, study-level, pooled) mixed effects model to best capture the variance observed in the data. In the event of significant main effects of any of our independent variables of interest, robust variance estimation (RVE) was then used to verify significance by including a standard error estimate into the selected model (see Eqs. 1–2).

#### Auditory Oddball

A comparison of models of auditory imaging effect sizes revealed that the full model was superior to the reduced model (p < 0.0001). The RVE-estimated pooled effect (z = 0.0032) reveals a negligible relationship between neural responses to auditory oddballs and diagnostic group. We then assessed if there were main effects of age, symptomology score, oddball discriminator, stimulus type, or imaging variable (e.g., MMN amplitude), publication year, task type (active or passive) or stimulus emotionality on effect size. We hypothesized that younger age and increased autism features would correspond with increased effect size, as would more complex auditory stimuli (speech sounds, syllabic oddballs, etc.). Additionally, due to prior findings of atypical auditory MMN/N2 latencies and amplitudes described previously, we expected that we would find a main effect of imaging variable, driven by MMN and N2 values. There were no significant main effects, so no further interaction effects were explored nor was RVE conducted.

When examining the auditory behavior effect sizes, we found no significant difference between the 2- and 3-level models, so the more parsimonious 2-level model was used. The estimated pooled effect (z = 0.144) revealed only a weak relationship between behavioral responses to auditory oddballs and diagnostic group. Although a main effect of symptomology score (p = 0.0115) emerged from the 2-level model, this analysis did not survive RVE (p = 0.974). No other moderators were significant.

Thus, from our analyses, differences in effect sizes across auditory oddball studies appear not to be related to participant or study characteristics, or stimulus features. We believe this finding may reflect the extreme population heterogeneity that is not sufficiently captured in autism research. Indeed, it is quite common for studies to engage autistic individuals with average or above average language abilities and intelligence quotients, which makes experimental compliance more likely, but which is not representative of the broader autism population. Indeed, the change in diagnostic criteria with the introduction of the DSM-5 (American Psychiatric Association, 2013) in 2013—which condensed autistic disorder, Asperger’s syndrome, and PDD-NOS into a singular diagnosis of autism spectrum disorder—has led to a significant increase in rates of autism diagnosis (Russell et al., 2022), but a significant decrease in overall effect sizes across meta-analyses (Rødgaard et al., 2019). While this particular confound is beyond the scope of this meta-analysis, we would be remiss not to acknowledge the potential impacts if the diluted effect sizes across autism research more broadly.

#### Visual Oddball

For visual EEG effect sizes, the full model was superior to the reduced model (p = 0.0004). The RVE-estimated pooled effect was negligible (z = 0.049). Hypotheses were the same as described in the prior section. We did observe main effects of oddball discriminator, publication year, and activity level (passive or active) on effect sizes, but none of these results retained significance after RVE was conducted.

For the studies with behavioral data, no emotional stimuli were used, so this moderator was not considered. We observed a trend toward significance in favor of the reduced model (p = 0.0545). Though the estimate of the pooled effect (z = 0.229) revealed only a weak relationship between behavioral performance on visual oddball tasks and diagnostic group, this relationship is stronger than that observed in the auditory behavioral model.

The same moderators as described above were evaluated in the visual models. While we observed a main effect of oddball discriminator, stimulus type, behavioral variable, and publication year, no significant effects were retained after RVE. Similarly, our interaction effects that were significant under the standard 2-level model (oddball discriminator × age; stimulus type × age; behavioral variable × age; behavioral variable x symptomology) did not retain their significance.

Therefore, as with the auditory analyses, differences in effect sizes across visual oddball studies are largely unrelated to participant characteristics or stimulus features. The notable exceptions are the interactions between oddball discriminator and age and stimulus type and age in EEG markers of change detection. However, as mentioned above, the lack of power makes it impossible to draw conclusions about group EEG differences at this time, including which stimulus types and oddball discriminators are likely to elicit the largest differences.

### Study Quality

The mean Selection of Participants score was 3.17 ± 1.09 (range 0–4.5; compare to average of 3.3 as described in Williams et al., 2023); the mean Participant Comparability score was 1.29 ± 0.52 (range 0–2; compare to average of 0.9 as described in Williams et al., 2023); only 23.08% of studies explicitly excluded syndromic autism, which reduced study quality. Total quality scores (mean 4.63 ± 1.65, range 0–7.5) were largely driven by the Selection of Participants component, with studies primarily being penalized for lack of sufficient IQ and age matching of control participants (see Supplementary Table 1, Item 3; mean = 0.56 ± 0.5 (range 0–1).

We were surprised that so few studies excluded syndromic autism (or failed to specify exclusion criteria for this). There are a variety of causes of syndromic autism (e.g., Fragile X Syndrome) that have distinctive additional clinical features which may artificially inflate effect size. Additionally, we found the lack of age matching problematic. Many of the studies used pediatric samples, and the degree of cognitive and neural change that occurs throughout childhood is staggering—in these samples, mismatched ages by even a year or 2 could potentially overshadow any real group differences.

## Discussion

### Comparable Change Detection in Auditory and Visual Oddball Tasks

Our chief finding is that there appear to be no significant differences in auditory or visual change detection in autism, even when accounting for participant or stimulus characteristics. Thus, our recommendation is to include auditory and visual oddball tasks in a battery of sensory prediction paradigms, rather than as the sole focus of a study. The exception to this recommendation is in the case of comparing simple and complex oddballs. We were not aware of any studies that directly compared neural and behavioral responses to oddballs of increasing complexity. This may be a fruitful approach, as research in other domains has shown that sensory processing differences are amplified as stimulus complexity increases (e.g., visual discrimination).

Indeed, when examining only studies that used complex social stimuli (speech, faces), we did observe larger effect sizes on the whole, except for visual behavioral effect sizes. This suggests that at the lowest levels of stimulus complexity, change detection in autism may be comparable to that of non-autistic individuals, but that deviance detection for complex social stimuli may evoke both behavioral and neural differences in this population. This suggestion comes with the caveat that while we found no evidence that the complexity of the stimulus had any effect on average study effect sizes, there were relatively few studies included in the meta-analysis, and more data is needed. Nonetheless, this finding is in line with other studies across a variety of domains that have shown the magnitude of group difference increases with growing stimulus complexity.

Relatedly, we explored the relationship between emotionality of the stimuli and effect size, and we were surprised to find none. Indeed, in healthy adults, both auditory and visual deviance responses are modulated by emotional valence. For instance, it has been observed that negative valence auditory deviants (e.g., cries) elicit larger MMN amplitudes relative to neutral deviants (Kao & Zhang, 2023; Ringer et al., 2024), perhaps suggesting additional attentional allocation to negatively coded auditory stimuli. Additionally, negative valence visual deviants produced enhanced delta and theta power compared to responses to neutral deviants (Bölükbaş et al., 2023), which traditionally may be viewed as augmented long-range network connectivity. Given that autistic individuals are known to exhibit changes to high-level cognitive functions (e.g., attention) and network connectivity, we would have expected some relationship to emerge.

From a computational standpoint, an increasing number of models support the hypothesis of weaker environmental expectations in autism (Pellicano & Burr, 2012; Zaidel et al., 2015; Van de Cruys et al., 2021; Noel et al., 2020; Park et al., 2017; Amoruso et al., 2019; Chambon et al., 2017), leading to less precise stimulus representations, and presumably in the context of oddball paradigms, reduced neural responses to change. That said, there are a variety of other computational theories that challenge this view (see, e.g., (Lidstone et al., 2023) for a comprehensive review of alternative hypotheses). For instance, rather than exhibiting *weaker* environmental expectations, the opposite may be true, with some autistic individuals exhibiting excessively *strong* expectations, which may result in inability to assimilate new information into an environmental schema, resulting in a slowness to habituate to stimuli and a need for more stimulus repeats to elicit characteristic change-related ERPs. Other accounts include broadly atypical prediction ability or changes to how certain modalities are weighted in relation to each other. While discussion of these alternative theories is beyond the scope of this review, they are worth noting.

While we found no study-level evidence to support any prediction-related hypothesis, exploration of change detection may reveal sensory prediction differences at the individual level: every individual is subject to their own sensory bias, noise, and priors that may lead to prediction errors. In this way, it may be beneficial to entertain a variety of models and compare their prevalence across autistic individuals.

**Fig. 5:**
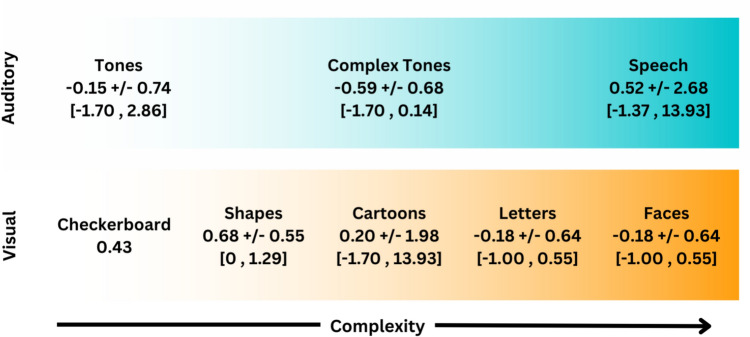
Schematic illustrating range of auditory (top) and visual (bottom) stimulus complexity. For each stimulus type, all average effect sizes for studies that included that stimulus were calculated and are shown above ± effect size standard deviation. Effect size range shown below in brackets

In sum, our results show that unisensory oddball paradigms are not likely to produce meaningful, true group level differences in autism, at least at the lowest levels of complexity. Furthermore, within and between stimulus types, we observe that there are both positive and negative effect sizes represented as well as large variabilities (Fig. 5). For example, while auditory oddball speech and complex tone paradigms both elicit robust pooled effect sizes, the standard deviation is larger than pooled effect size in both cases, and effect sizes are in opposite directions. Thus, it is unclear what neural mechanisms may underlie these group differences, if any. The visual domain is even more puzzling, with no clear pattern emerging in mean effect size or range.

If sensory prediction truly is affected in autism, as suggested by numerous studies and described above, it seems that extant oddball paradigms are not capturing this effectively. Perhaps oddball paradigms are sufficiently “predictable” or “simple” that models of prediction in autism do not apply. These models do frequently examine environmental expectations in the context of naturalistic stimuli and are often described in terms of conceptual or categorial expectations, rather than at the level of stimulus features. Thus, we may expect that such models do not apply to deviant stimuli unless the *meaning* of the stimulus changes (e.g., speech, faces, etc.).

We recommend that oddball studies in the autism population be used in conjunction with other paradigms examining either change detection or the cascading effects of stimulus complexity. For example, a visual oddball paradigm using a simple checkerboard stimulus may be paired with a masked motion paradigm using the same stimulus to examine prediction across different contexts. In contrast, auditory and visual oddball paradigms may be conducted using stimuli across a variety of stimulus complexities within the same individual, or even paired with multisensory stimuli. A cursory search shows no use of audiovisual oddball paradigms in autism research, which may yield significant and more meaningful results that mimic ecologically valid scenarios (e.g., audiovisual speech oddball).

### Diversity in Autism must be Characterized

It is also important to note that opposing findings between studies—and indeed, in numerous domains of autism research—may also be related to participant characteristics. It is a well known problem in the field that it is very difficult to generalize findings across the population. In our view, the primary reason for this is the expansive spectrum of behaviors encapsulated within the autism spectrum diagnosis. Furthermore, because autism is a neurodevelopmental condition, the significant changes that occur throughout an autistic individual’s lifespan make it difficult to even characterize the degree of clinical presentation conclusively—many autism features will be “outgrown” or change in their presentation as a child ages. A secondary reason is that autism research heavily depends on participation by verbal, low-support individuals, while autistic individuals with high support needs—often including those with syndromic autism—are drastically under-represented.

The studies included in this meta-analysis ranked poorly according to NOS scoring standards, largely in part due to lack of age and IQ matching between autistic and control participants, as well as lack of syndromic autism exclusion. While we as autism researchers ourselves understand the challenge of matching children by age group, we suggest that future studies—not just oddball paradigms—more rigorously adhere to these practices. Additionally, while we do believe that autism research should be more inclusive of syndromic autism, it is concerning that many studies simply did not specify the syndromes included in the autistic sample. It is our belief that a greater effort should be made to understand how autism phenotypes—and known genotypes—cluster together, rather than declaring research results about autism more broadly.

### Limitations

As noted above, there was a dearth of visual oddball studies, which made it challenging to draw more robust conclusions about the nature of visual prediction in autism. Additionally, many of these studies did not have participant characteristics readily available, nor did many authors respond to requests for information upon inquiry. Thus, we are limited in our ability to determine whether there is a significant relationship between clinical autism features and sensory prediction.

## Supplementary Information

Below is the link to the electronic supplementary material.

Supplementary file 1 (XLSX 106 kb)

Supplementary file 2 (DOCX 114 kb)

## References

[CR1] American Psychiatric Association. (2013). *Diagnostic and statistical manual of mental disorders* (5th ed.). American Psychiatric Association.

[CR2] Amoruso, L., Narzisi, A., Pinzino, M., Finisguerra, A., Billeci, L., Calderoni, S., Fabbro, F., Muratori, F., Volzone, A., & Urgesi, C. (2019). Contextual priors do not modulate action prediction in children with autism. *Proceedings of the Royal Society B,**286*(1908), 20191319. 31409253 10.1098/rspb.2019.1319 PMC6710602

[CR3] Arthur, T., Vine, S., Buckingham, G., Brosnan, M., Wilson, M., & Harris, D. (2023). Testing predictive coding theories of autism spectrum disorder using models of active inference. *PLOS Computational Biology,**19*(9), e1011473. 10.1371/journal.pcbi.1011473 37695796 10.1371/journal.pcbi.1011473 PMC10529610

[CR4] Balduzzi, S., Rücker, G., & Schwarzer, G. (2019). How to perform a meta-analysis with R: A practical tutorial. *Evidence-Based Mental Health,**22*, 153–160. 10.1136/ebmental-2019-300117 31563865 10.1136/ebmental-2019-300117 PMC10231495

[CR5] Baranek, G. T., Woynaroski, T. G., Nowell, S., Turner-Brown, L., DuBay, M., Crais, E. R., & Watson, L. R. (2018). Cascading effects of attention disengagement and sensory seeking on social symptoms in a community sample of infants at-risk for a future diagnosis of autism spectrum disorder. *Autism Spectrum Condition Understanding Sensory and Social Features*, *29*, 30–40. 10.1016/j.dcn.2017.08.006 10.1016/j.dcn.2017.08.006 PMC6414208 28869201

[CR6] Bölükbaş, B., Aktürk, T., Ardalı, H., Dündar, Y., Güngör, C., Kahveci, Ş, & Güntekin, B. (2023). Event-related delta and theta responses may reflect the valence discrimination in the emotional oddball task. *Cognitive Processing,**24*(4), 595–608. 10.1007/s10339-023-01158-w 37615788 10.1007/s10339-023-01158-w

[CR7] Bonnel, A., McAdams, S., Smith, B., Berthiaume, C., Bertone, A., Ciocca, V., Burack, J. A., & Mottron, L. (2010). Enhanced pure-tone pitch discrimination among persons with autism but not Asperger syndrome. *Neuropsychologia,**48*(9), 2465–2475. 10.1016/j.neuropsychologia.2010.04.020 20433857 10.1016/j.neuropsychologia.2010.04.020

[CR8] Boyd, B. A., Baranek, G. T., Sideris, J., Poe, M. D., Watson, L. R., Patten, E., & Miller, H. (2010). Sensory features and repetitive behaviors in children with autism and developmental delays. *Autism Research,**3*(2), 78–87. 10.1002/aur.124 20437603 10.1002/aur.124 PMC3071028

[CR9] Brooks, P. J., Gaggi, N. L., & Ploog, B. O. (2018). Generalization of content and emotional prosody across speakers varying in gender in youth with Autism Spectrum Disorder. *Research in Developmental Disabilities,**83*, 57–68. 10.1016/j.ridd.2018.08.004 30142574 10.1016/j.ridd.2018.08.004

[CR10] Callan, D. E., Jones, J. A., Munhall, K., Callan, A. M., Kroos, C., & Vatikiotis-Bateson, E. (2003). Neural processes underlying perceptual enhancement by visual speech gestures. *NeuroReport*, *14*(17). https://journals.lww.com/neuroreport/Fulltext/2003/12020/Neural_processes_underlying_perceptual_enhancement.16.aspx 10.1097/00001756-200312020-00016 14625450

[CR11] Calvert, G. A., & Campbell, R. (2003). Reading speech from still and moving faces: The neural substrates of visible speech. *Journal of Cognitive Neuroscience,**15*(1), 57–70. 10.1162/089892903321107828 12590843 10.1162/089892903321107828

[CR12] Cary, E., Pacheco, D., Kaplan-Kahn, E., McKernan, E., Matsuba, E., Prieve, B., & Russo, N. (2024). Brain signatures of early and late neural measures of auditory habituation and discrimination in autism and their relationship to autistic traits and sensory overresponsivity. *Journal of Autism and Developmental Disorders,**54*(4), 1344–1360. 10.1007/s10803-022-05866-8 36626009 10.1007/s10803-022-05866-8

[CR13] Chambon, V., Farrer, C., Pacherie, E., Jacquet, P. O., Leboyer, M., & Zalla, T. (2017). Reduced sensitivity to social priors during action prediction in adults with autism spectrum disorders. *Cognition,**160*, 17–26. 10.1016/j.cognition.2016.12.005 28039782 10.1016/j.cognition.2016.12.005

[CR14] Courchesne, E., Kilman, B. A., Galambos, R., & Lincoln, A. J. (1984). Autism: Processing of novel auditory information assessed by event-related brain potentials. *Electroencephalography and Clinical Neurophysiology/Evoked Potentials Section,**59*(3), 238–248. 10.1016/0168-5597(84)90063-7 10.1016/0168-5597(84)90063-7 6203714

[CR15] Courchesne, E., Townsend, J., Akshoomoff, N. A., Saitoh, O., Yeung-Courchesne, R., Lincoln, A. J., James, H. E., Haas, R. H., Schreibman, L., & Lau, L. (1994). Impairment in shifting attention in autistic and cerebellar patients. *Behavioral Neuroscience,**108*(5), 848–865. 10.1037/0735-7044.108.5.848 7826509 10.1037//0735-7044.108.5.848

[CR16] Courchesne, E., & Allen, G. (1997). Prediction and preparation, fundamental functions of the cerebellum. *Learning Memory*, *4*(1), 1–35. 10.1101/lm.4.1.1 10456051 10.1101/lm.4.1.1

[CR17] Criel, Y., Boon, C., Depuydt, E., Stalpaert, J., Huysman, E., Miatton, M., Santens, P., van Mierlo, P., & De Letter, M. (2023). Aging and sex effects on phoneme perception: An exploratory mismatch negativity and P300 investigation. *International Journal of Psychophysiology,**190*, 69–83. 10.1016/j.ijpsycho.2023.06.002 37301445 10.1016/j.ijpsycho.2023.06.002

[CR18] Crosse, M. J., Butler, J. S., & Lalor, E. C. (2015). Congruent visual speech enhances cortical entrainment to continuous auditory speech in noise-free conditions. *The Journal of Neuroscience,**35*(42), 14195. 10.1523/JNEUROSCI.1829-15.2015 26490860 10.1523/JNEUROSCI.1829-15.2015 PMC6605423

[CR20] Dwyer, P., Williams, Z. J., Vukusic, S., Saron, C. D., & Rivera, S. M. (2023). Habituation of auditory responses in young autistic and neurotypical children. *Autism Research,**16*(10), 1903–1923. 10.1002/aur.3022 37688470 10.1002/aur.3022 PMC10651062

[CR21] Egger, M., Smith, G. D., Schneider, M., & Minder, C. (1997). Bias in meta-analysis detected by a simple, graphical test. *BMJ,**315*(7109), 629–634. 9310563 10.1136/bmj.315.7109.629 PMC2127453

[CR22] Fisher, Z. F., & Tipton, E. (2015). robumeta: An R-package for robust variance estimation in meta-analysis. *arXiv: Methodology*. https://api.semanticscholar.org/CorpusID:46908133

[CR23] Foss-Feig, J. H., Heacock, J. L., & Cascio, C. J. (2012). Tactile responsiveness patterns and their association with core features in autism spectrum disorders. *Research in Autism Spectrum Disorders,**6*(1), 337–344. 10.1016/j.rasd.2011.06.007 22059092 10.1016/j.rasd.2011.06.007 PMC3207504

[CR24] Friston, K. (2009). The free-energy principle: A rough guide to the brain? *Trends in Cognitive Sciences,**13*(7), 293–301. 10.1016/j.tics.2009.04.005 19559644 10.1016/j.tics.2009.04.005

[CR25] Grillon, C., Courchesne, E., & Akshoomoff, N. (1989). Brainstem and middle latency auditory evoked potentials in autism and developmental language disorder. *Journal of Autism and Developmental Disorders,**19*(2), 255–269. 10.1007/BF02211845 2745391 10.1007/BF02211845

[CR26] Harrer, M., Cuijpers, P., Furukawa, T., & Ebert, D. (2019). *dmetar: Companion R Package For The Guide “Doing Meta-Analysis in R”* (Version 0.0.9000) [Computer software]. http://dmetar.protectlab.org/

[CR27] Heaton, P. (2003). Pitch memory, labelling and disembedding in autism. *Journal of Child Psychology and Psychiatry,**44*(4), 543–551. 10.1111/1469-7610.00143 12751846 10.1111/1469-7610.00143

[CR28] Heaton, P., Williams, K., Cummins, O., & Happe, F. (2008). Autism and pitch processing splinter skills: A group and subgroup analysis. *Autism,**12*(2), 203–219. 18308768 10.1177/1362361307085270

[CR29] Hedges, L. V., Tipton, E., & Johnson, M. C. (2010). Robust variance estimation in meta-regression with dependent effect size estimates. *Research Synthesis Methods,**1*(1), 39–65. 10.1002/jrsm.5 26056092 10.1002/jrsm.5

[CR30] Hocking, D. R., Sun, X., Haebich, K., Darke, H., North, K. N., Vivanti, G., & Payne, J. M. (2023). Delineating visual habituation profiles in preschoolers with neurofibromatosis Type 1 and autism spectrum disorder: A cross-syndrome study. *Journal of Autism and Developmental Disorders*. 10.1007/s10803-023-05913-y 36877426 10.1007/s10803-023-05913-y

[CR31] Hudac, C. M., DesChamps, T. D., Arnett, A. B., Cairney, B. E., Ma, R., Webb, S. J., & Bernier, R. A. (2018). Early enhanced processing and delayed habituation to deviance sounds in autism spectrum disorder. *Brain and Cognition,**123*, 110–119. 29550506 10.1016/j.bandc.2018.03.004 PMC5893357

[CR32] Kamp, S.-M., Forester, G., Vatheuer, C. C., & Domes, G. (2021). Stress effects on the oddball P300 and N2 in males and females. *Biological Psychology,**162*, 108095. 10.1016/j.biopsycho.2021.108095 33872742 10.1016/j.biopsycho.2021.108095

[CR33] Kao, C., & Zhang, Y. (2023). Detecting emotional prosody in real words: Electrophysiological evidence from a modified multifeature oddball paradigm. *Journal of Speech, Language, and Hearing Research,**66*(8), 2988–2998. 10.1044/2023_JSLHR-22-00652 37379567 10.1044/2023_JSLHR-22-00652

[CR34] Korpilahti, P., Jansson-Verkasalo, E., Mattila, M.-L., Kuusikko, S., Suominen, K., Rytky, S., Pauls, D. L., & Moilanen, I. (2007). Processing of affective speech prosody is impaired in Asperger syndrome. *Journal of Autism and Developmental Disorders,**37*(8), 1539–1549. 10.1007/s10803-006-0271-2 17086440 10.1007/s10803-006-0271-2

[CR35] Lane, A. E., Young, R. L., Baker, A. E. Z., & Angley, M. T. (2010). Sensory processing subtypes in autism: Association with adaptive behavior. *Journal of Autism and Developmental Disorders,**40*(1), 112–122. 10.1007/s10803-009-0840-2 19644746 10.1007/s10803-009-0840-2

[CR36] Lidstone, D. E., Mostofsky, S. H., & Ewen, J. (2023). *Towards experimental approaches to advance discovery of clinically meaningful sensory-motor biomarkers*. PsyArXiv. 10.31234/osf.io/hxd67

[CR37] Ma, W. J., Zhou, X., Ross, L. A., Foxe, J. J., & Parra, L. C. (2009). Lip-reading aids word recognition most in moderate noise: A Bayesian explanation using high-dimensional feature space. *PLoS ONE,**4*(3), e4638. 10.1371/journal.pone.0004638 19259259 10.1371/journal.pone.0004638 PMC2645675

[CR38] Minshew, N. J., & Goldstein, G. (1998). Autism as a disorder of complex information processing. *Mental Retardation and Developmental Disabilities Research Reviews,**4*(2), 129–136. 10.1002/(SICI)1098-2779(1998)4:2<129::AID-MRDD10>3.0.CO;2-X

[CR39] Minshew, N. J., Goldstein, G., & Siegel, D. J. (1997). Neuropsychologic functioning in autism: Profile of a complex information processing disorder. *Journal of the International Neuropsychological Society,**3*(4), 303–316. 10.1017/S1355617797003032 9260440

[CR40] Morr, M. L., Shafer, V. L., Kreuzer, J. A., & Kurtzberg, D. (2002). Maturation of mismatch negativity in typically developing infants and preschool children. *Ear and Hearing*, *23*(2). https://journals.lww.com/ear-hearing/fulltext/2002/04000/maturation_of_mismatch_negativity_in_typically.5.aspx 10.1097/00003446-200204000-00005 11951848

[CR41] Möttönen, R., Krause, C. M., Tiippana, K., & Sams, M. (2002). Processing of changes in visual speech in the human auditory cortex. *Cognitive Brain Research,**13*(3), 417–425. 10.1016/S0926-6410(02)00053-8 11919005 10.1016/s0926-6410(02)00053-8

[CR42] Näätänen, R., Paavilainen, P., Rinne, T., & Alho, K. (2007). The mismatch negativity (MMN) in basic research of central auditory processing: A review. *Clinical Neurophysiology,**118*(12), 2544–2590. 10.1016/j.clinph.2007.04.026 17931964 10.1016/j.clinph.2007.04.026

[CR43] Noel, J.-P., Lakshminarasimhan, K. J., Park, H., & Angelaki, D. E. (2020). Increased variability but intact integration during visual navigation in Autism Spectrum Disorder. *Proceedings of the National Academy of Sciences,**117*(20), 11158. 10.1073/pnas.2000216117 10.1073/pnas.2000216117 PMC7245105 32358192

[CR44] Palmer, C. J., Lawson, R. P., & Hohwy, J. (2017). Bayesian approaches to autism: Towards volatility, action, and behavior. *Psychological Bulletin,**143*(5), 521. 28333493 10.1037/bul0000097

[CR45] Park, W. J., Schauder, K. B., Zhang, R., Bennetto, L., & Tadin, D. (2017). High internal noise and poor external noise filtering characterize perception in autism spectrum disorder. *Scientific Reports,**7*(1), 1–12. 29242499 10.1038/s41598-017-17676-5 PMC5730555

[CR46] Pellicano, E., & Burr, D. (2012). When the world becomes ‘too real’: A Bayesian explanation of autistic perception. *Trends in Cognitive Sciences,**16*(10), 504–510. 10.1016/j.tics.2012.08.009 22959875 10.1016/j.tics.2012.08.009

[CR47] Plass, J., Brang, D., Suzuki, S., & Grabowecky, M. (2020). Vision perceptually restores auditory spectral dynamics in speech. *Proceedings of the National Academy of Sciences of the United States of America,**117*(29), 16920–16927. 10.31234/osf.io/t954p 32632010 10.1073/pnas.2002887117 PMC7382243

[CR48] Polich, J. (2007). Updating P300: An integrative theory of P3a and P3b. *Clinical Neurophysiology,**118*(10), 2128–2148. 10.1016/j.clinph.2007.04.019 17573239 10.1016/j.clinph.2007.04.019 PMC2715154

[CR49] Polich, J., Howard, L., & Starr, A. (1985). Stimulus frequency and masking as determinants of P300 latency in event-related potentials from auditory stimuli. *Biological Psychology,**21*(4), 309–318. 4096911 10.1016/0301-0511(85)90185-1

[CR50] Pustejovsky, J. (2022). *_clubSandwich: Cluster-Robust (Sandwich) Variance Estimators with Small-Sample Corrections_* (Version 0.5.8) [Computer software].

[CR51] R Core Team. (2017). *R: A language and environment for statistical computing* [Computer software]. R Foundation for Statistical Computing. https://www.R-project.org/

[CR52] Ringer, H., Rösch, S. A., Roeber, U., Deller, J., Escera, C., & Grimm, S. (2024). That sounds awful! Does sound unpleasantness modulate the mismatch negativity and its habituation? *Psychophysiology,**61*(2), e14450. 10.1111/psyp.14450 37779371 10.1111/psyp.14450

[CR53] Ritter, W., Simson, R., Vaughan, H. G., & Friedman, D. (1979). A brain event related to the making of a sensory discrimination. *Science,**203*(4387), 1358–1361. 10.1126/science.424760 424760 10.1126/science.424760

[CR54] Rivest, J. B., Jemel, B., Bertone, A., McKerral, M., & Mottron, L. (2013). Luminance- and texture-defined information processing in school-aged children with autism. *PLoS ONE,**8*(10), e78978. 10.1371/journal.pone.0078978 24205355 10.1371/journal.pone.0078978 PMC3812000

[CR55] Rødgaard, E.-M., Jensen, K., Vergnes, J.-N., Soulières, I., & Mottron, L. (2019). Temporal changes in effect sizes of studies comparing individuals with and without autism: A meta-analysis. *JAMA Psychiatry,**76*(11), 1124–1132. 10.1001/jamapsychiatry.2019.1956 31433441 10.1001/jamapsychiatry.2019.1956 PMC6704749

[CR56] Rosenblum, L. D., Johnson, J. A., & Saldaña, H. M. (1996). Point-light facial displays enhance comprehension of speech in noise. *Journal of Speech, Language, and Hearing Research,**39*(6), 1159–1170. 10.1044/jshr.3906.1159 10.1044/jshr.3906.1159 8959601

[CR57] Ross, L. A., Saint-Amour, D., Leavitt, V. M., Javitt, D. C., & Foxe, J. J. (2007). Do you see what i am saying? Exploring visual enhancement of speech comprehension in noisy environments. *Cerebral Cortex,**17*(5), 1147–1153. 10.1093/cercor/bhl024 16785256 10.1093/cercor/bhl024

[CR58] Rugg, M. D., & Coles, M. G. (1995). *Electrophysiology of mind: Event-related brain potentials and cognition.* Oxford University Press.

[CR59] Ruiz-Martínez, F. J., Rodríguez-Martínez, E. I., Wilson, C. E., Yau, S., Saldaña, D., & Gómez, C. M. (2020). Impaired P1 habituation and mismatch negativity in children with autism spectrum disorder. *Journal of Autism and Developmental Disorders,**50*(2), 603–616. 10.1007/s10803-019-04299-0 31728809 10.1007/s10803-019-04299-0

[CR60] Russell, G., Stapley, S., Newlove-Delgado, T., Salmon, A., White, R., Warren, F., Pearson, A., & Ford, T. (2022). Time trends in autism diagnosis over 20 years: A UK population-based cohort study. *Journal of Child Psychology and Psychiatry,**63*(6), 674–682. 10.1111/jcpp.13505 34414570 10.1111/jcpp.13505

[CR61] Sams, M., Aulanko, R., Hämäläinen, M., Hari, R., Lounasmaa, O. V., Lu, S.-T., & Simola, J. (1991). Seeing speech: Visual information from lip movements modifies activity in the human auditory cortex. *Neuroscience Letters,**127*(1), 141–145. 10.1016/0304-3940(91)90914-F 1881611 10.1016/0304-3940(91)90914-f

[CR62] Samson, F., Mottron, L., Jemel, B., Belin, P., & Ciocca, V. (2006). Can spectro-temporal complexity explain the autistic pattern of performance on auditory tasks? *Journal of Autism and Developmental Disorders,**36*(1), 65–76. 10.1007/s10803-005-0043-4 16382329 10.1007/s10803-005-0043-4

[CR63] Schulz, S. E., & Stevenson, R. A. (2019). Sensory hypersensitivity predicts repetitive behaviours in autistic and typically-developing children. *Autism,**23*(4), 1028–1041. 10.1177/1362361318774559 30244585 10.1177/1362361318774559

[CR64] Sinha, P., Kjelgaard, M. M., Gandhi, T. K., Tsourides, K., Cardinaux, A. L., Pantazis, D., Diamond, S. P., & Held, R. M. (2014). Autism as a disorder of prediction. *Proceedings of the National Academy of Sciences,**111*(42), 15220–15225. 10.1073/pnas.1416797111 10.1073/pnas.1416797111 PMC4210351 25288765

[CR65] Sumby, W. H., & Pollack, I. (1954). Visual contribution to speech intelligibility in noise. *The Journal of the Acoustical Society of America,**26*(2), 212–215. 10.1121/1.1907309

[CR66] Tarasi, L., Trajkovic, J., Diciotti, S., di Pellegrino, G., Ferri, F., Ursino, M., & Romei, V. (2022). Predictive waves in the autism-schizophrenia continuum: A novel biobehavioral model. *Neuroscience & Biobehavioral Reviews,**132*, 1–22. 10.1016/j.neubiorev.2021.11.006 34774901 10.1016/j.neubiorev.2021.11.006

[CR67] Tomé, D., Barbosa, F., Nowak, K., & Marques-Teixeira, J. (2015). The development of the N1 and N2 components in auditory oddball paradigms: A systematic review with narrative analysis and suggested normative values. *Journal of Neural Transmission,**122*(3), 375–391. 10.1007/s00702-014-1258-3 24961573 10.1007/s00702-014-1258-3

[CR68] Townsend, J., Harris, N. S., & Courchesne, E. (1996). Visual attention abnormalities in autism: Delayed orienting to location. *Journal of the International Neuropsychological Society*, *2*(6), 541–550. 10.1017/S1355617700001715 10.1017/s1355617700001715 9375158

[CR69] Van de Cruys, S., Evers, K., Van der Hallen, R., Van Eylen, L., Boets, B., & de Wit, L., & Wagemans, J. (2014). Precise minds in uncertain worlds: Predictive coding in autism. *Psychological Review,**121*(4), 649–675. 10.1037/a0037665 25347312 10.1037/a0037665

[CR19] Van de Cruys, S., Lemmens, L., Sapey-Triomphe, L.-A., Chetverikov, A., Noens, I., & Wagemans, J. (2021). Structural and contextual priors affect visual search in children with and without autism. *Autism Research*, *n/a*(n/a). 10.1002/aur.2511 10.1002/aur.2511 33811474

[CR70] van Dinteren, R., Arns, M., Jongsma, M. L. A., & Kessels, R. P. C. (2014). P300 development across the Lifespan: A systematic review and meta-analysis. *PLoS ONE,**9*(2), e87347. 10.1371/journal.pone.0087347 24551055 10.1371/journal.pone.0087347 PMC3923761

[CR71] van Wassenhove, V., Grant, K. W., & Poeppel, D. (2005). Visual speech speeds up the neural processing of auditory speech. *Proceedings of the National Academy of Sciences of the United States of America,**102*(4), 1181. 10.1073/pnas.0408949102 15647358 10.1073/pnas.0408949102 PMC545853

[CR72] Viechtbauer, W. (2010). Conducting meta-analyses in R with the metafor package. *Journal of Statistical Software*, *36*(3), 1–48. 10.18637/jss.v036.i03

[CR73] Vogel, E. K., & Luck, S. J. (2000). The visual N1 component as an index of a discrimination process. *Psychophysiology,**37*(2), 190–203. 10.1111/1469-8986.3720190 10731769

[CR74] von der Lühe, T., Manera, V., Barisic, I., Becchio, C., Vogeley, K., & Schilbach, L. (2016). Interpersonal predictive coding, not action perception, is impaired in autism. *Philosophical Transactions of the Royal Society B: Biological Sciences,**371*(1693), 20150373. 10.1098/rstb.2015.0373 10.1098/rstb.2015.0373 PMC4843611 27069050

[CR75] Webb, S. J., Jones, E. J. H., Merkle, K., Namkung, J., Toth, K., Greenson, J., Murias, M., & Dawson, G. (2010). Toddlers with elevated autism symptoms show slowed habituation to faces. *Child Neuropsychology,**16*(3), 255–278. 10.1080/09297041003601454 20301009 10.1080/09297041003601454 PMC2989718

[CR76] Wells, G. A., Wells, G., Shea, B., Shea, B., O’Connell, D., Peterson, J., Welch, Losos, M., Tugwell, P., Ga, S. W., Zello, G. A., & Petersen, J. A. (2014). The Newcastle-Ottawa Scale (NOS) for assessing the quality of nonrandomised studies in meta-analyses. https://api.semanticscholar.org/CorpusID:79550924

[CR77] Williams, Z. J., Suzman, E., Bordman, S. L., Markfeld, J. E., Kaiser, S. M., Dunham, K. A., Zoltowski, A. R., Failla, M. D., Cascio, C. J., & Woynaroski, T. G. (2023). Characterizing interoceptive differences in autism: A systematic review and meta-analysis of case–control studies. *Journal of Autism and Developmental Disorders,**53*(3), 947–962. 10.1007/s10803-022-05656-2 35819587 10.1007/s10803-022-05656-2 PMC9832174

[CR78] Wilson, D. B. (2023). *Practical meta-analysis effect size calculator * (version 2023.11.27)

[CR79] Zaidel, A., Goin-Kochel, R. P., & Angelaki, D. E. (2015). Self-motion perception in autism is compromised by visual noise but integrated optimally across multiple senses. *Proceedings of the National Academy of Sciences,**112*(20), 6461–6466. 10.1073/pnas.1506582112 10.1073/pnas.1506582112 PMC4443344 25941373

