# Supplementary material for: Minimal Differences in Auditory and Visual Oddball Tasks in Autism: A Systematic Review and Meta-Analysis
Source: J Autism Dev Disord. 2025 Mar 8;56(8):3099–113. doi: 10.1007/s10803-025-06772-5 (PMC13391720; doi:10.1007/s10803-025-06772-5)
Supplement: Supplementary file 2 — (DOCX 114 kb) [file 10803_2025_6772_MOESM2_ESM.docx]

***Supplementary Information***

| Supplementary Table 1. Adapted NOS Quality Metrics | |
| --- | --- |
| Selection of Participants | |
| Item 1. Is autism characterization adequate? | - 0.5 points if characterized with one standardized assessment (ADOS and/or ADI and/or CARS) in a majority of autistic participants (80% and over) - 0.5 points if characterized with standardized assessment + clinical judgment. “Clinical judgment” can be clinical interview, best estimate diagnosis, team of professionals - 0.5 points if comorbidities and/or medications taken by participants in autistic group are reported |
| Item 2. Are autism cases representative? | - 1 point if IQ range of 40+ or SD of 12+ among autistic participants on at least one IQ measure |
| Item 3. Are controls adequately selected? | - 1 point if attempt was made to recruit groups similar in terms of IQ and age (attempt at matching, no matter if in the end there is a group difference on one of these variables) |
| Item 4. Are controls adequately defined? | - 1 point if controls have no history of autism previously diagnosed. Examples may include a questionnaire/interview asking about neurological conditions and/or psychiatric conditions and/or developmental conditions; exclusion criteria mention autism and/or neurological/psychiatric/developmental conditions |
| **Selection of Participants = Sum of Items 1-4 (Range 0 - 4.5)** | |
| Participant Comparability | |
| Item 5. Are cases and controls matched*? | - 1 point if matched on intelligence (any test) or developmental level (e.g., Mullen Scales of Early Learning) - 0.5 points if matched on gender/sex - 0.5 points if matched on chronological age   *Matched means no significant between-group difference on the variable (if statistical test not reported, group means should be obviously close, within 0.25 SD) |
| **Participant Comparability = Item 5 Total (Range 0 - 2)** | |
| Exclusion of Syndromic Autism | |
| Item 6. Is syndromic autism excluded? | - 1 point if autistic participants with a known genetic condition or neurological conditions were excluded |
| **Exclusion of Syndromic Autism = Item 6 Total (Range 0 – 1)** | |
| **Total Study Quality = Sum of Items 1-6 (Range 0 – 7.5)** | |

| Supplementary Table 2. Study Characteristics | | | | | | | | | |
| --- | --- | --- | --- | --- | --- | --- | --- | --- | --- |
| **Author** | **Publication Year** | **Total N** | **Mean Age** | **Modality** | **Oddball Type** | **Stimulus Type** | **Hedges G** | **Variance** | **Contains Behavioral Data** |
| Courchesne et al., 1984^*^ | 1984 | 14 |  | auditory | semantic | speech | 1.32 | 0.232 | No |
| Erwin et al., 1991^*^ | 1991 | 25 | 25.45 | auditory | consonant, semantic, emotion | speech | 0.609 | 0.137 | No |
| Gomot et al., 2002 | 2002 | 30 | 6.79 | auditory | frequency | tone | 0.097 | 0.169 | No |
| Čeponienė et al., 2003* | 2003 | 19 | 8.65 | auditory | frequency | complex tone, tone | -0.752 | 0.139 | No |
| Ferri et al., 2003* | 2003 | 20 | 12.25 | auditory | frequency | tone | 2.855 | 0.372 | No |
| Gomot et al., 2002 | 2003 | 30 | 6.79 | auditory | frequency | tone | 0.155 | 0.167 | No |
| Jansson-Verkasalo et al., 2003 | 2003 | 21 | 9.35 | auditory | consonant, frequency | synthetic speech, tone | -0.134 | 0.239 | No |
| Kuhl et al., 2005 | 2004 | 44 | 3.91 | auditory | consonant | speech | 0 | 0.076 | No |
| Jansson-Verkasalo et al., 2005 | 2005 | 37 | 10.5 | auditory | frequency | tone | -0.102 | 0.172 | No |
| Kasai et al., 2005 | 2005 | 28 | 27.25 | auditory | frequency, duration, vowel | tone, speech | -0.029 | 0.132 | No |
| Kujala et al., 2005^*^ | 2005 | 16 | 32.5 | auditory | semantic | speech | -0.659 | 0.046 | Yes |
| Lepistö et al., 2005 | 2005 | 30 | 9.4 | auditory | vowel, frequency | speech, complex tone | -0.082 | 0.062 | No |
| Oram Cardy et al., 2005 | 2005 | 16 | 11.9 | auditory | vowel | speech | -0.372 | 0.128 | No |
| Gomot et al., 2006^*^ | 2006 | 24 | 13.65 | auditory | frequency | tone | -0.658 | 0.1 | No |
| Lepistö et al., 2006* | 2006 | 20 | 8.11 | auditory | frequency, duration, vowel | speech, complex tone | -1.369 | 0.124 | Yes |
| Korpilahti et al., 2007 | 2007 | 27 | 11 | auditory | frequency | speech | -0.413 | 0.077 | No |
| Kujala et al., 2007^*^ | 2007 | 18 | 28.5 | auditory | frequency, duration, gap, intensity, location | tone | -1.456 | 0.216 | No |
| Lepistö et al., 2007^*^ | 2007 | 18 | 28.5 | auditory | duration, frequency, vowel | speech, synthetic speech | 5.443 | 0.72 | Yes |
| Dunn et al., 2008 | 2008 | 36 | 9.33 | auditory | frequency | tone | -0.393 | 0.19 | No |
| Gomot et al., 2008 | 2008 | 24 | 13.65 | auditory | frequency | complex tone | -0.071 | 0.163 | Yes |
| Lepistö et al., 2008 | 2008 | 26 | 9.05 | auditory | frequency, consonant, vowel | synthetic speech | -0.417 | 0.293 | No |
| Whitehouse et al., 2009 | 2008 | 30 | 10.5 | auditory | vowel | speech | 0.166 | 0.27 | No |
| Kujala et al., 2010 | 2010 | 28 | 10.64 | auditory | vowel, frequency, duration, intensity, consonant | speech | -0.014 | 0.232 | No |
| Gomot et al., 2011^*^ | 2011 | 54 | 8.33 | auditory | frequency | tone | 0.383 | 0.113 | No |
| Guiraud et al., 2011 | 2011 | 56 | 0.76 | auditory | frequency | tone, complex tone | 0.135 | 0.153 | No |
| Roberts et al., 2011 | 2011 | 52 | 9.62 | auditory | frequency | tone | 0.39 | 0.198 | No |
| Fan & Cheng, 2014 | 2014 | 40 | 21.75 | auditory | vowel, frequency | speech, complex tone | -0.069 | 0.079 | No |
| Iwanami et al., 2014^*^ | 2014 | 37 | 29.35 | auditory | frequency | tone | 0.504 | 0.105 | Yes |
| Ludlow et al., 2014 | 2014 | 22 | 13.35 | auditory | semantic | speech | -0.41 | 0.087 | No |
| Abdeltawwab & Baz, 2015 | 2015 | 61 | 11.29 | auditory | frequency | tone | -0.363 | 0.218 | No |
| Donkers et al., 2015* | 2015 | 67 | 7.32 | auditory | frequency, duration | tone | 1.459 | 0.35 | No |
| Gonzalez-Gadea et al., 2015^*^ | 2015 | 43 | 11.01 | auditory | frequency | complex tone | -1.702 | 0.224 | No |
| Lindström et al., 2016 | 2016 | 23 | 10.25 | auditory | semantic | speech | -0.046 | 0.162 | No |
| Matsuzaki et al., 2017^*^ | 2017 | 33 | 9.27 | auditory | frequency | complex tone | -0.79 | 0.184 | No |
| Vlaskamp et al., 2017 | 2017 | 73 | 11 | auditory | frequency | tone | -0.255 | 0.115 | No |
| X. Wang et al., 2017 | 2017 | 31 | 10.35 | auditory | vowel, frequency | speech, tone | -0.115 | 0.205 | No |
| Yoshimura et al., 2017 | 2017 | 93 | 4.94 | auditory | semantic | speech | -0.385 | 0.164 | No |
| Charpentier et al., 2018 | 2018 | 28 | 9.9 | auditory | vowel | speech | -0.078 | 0.131 | No |
| Goris et al., 2018* | 2018 | 54 | 32.78 | auditory | frequency | tone | -0.782 | 0.144 | No |
| Huang et al., 2018 | 2018 | 28 | 9.55 | auditory | frequency, vowel | tone, speech | -0.084 | 0.104 | No |
| Hudac et al., 2018 | 2018 | 133 | 12.78 | auditory | frequency | tone | 0.053 | 0.099 | No |
| Lindström et al., 2018 | 2018 | 31 | 10.25 | auditory | frequency | speech | -0.129 | 0.101 | Yes |
| Nijhof et al., 2018 | 2018 | 48 | 32.15 | auditory | semantic | one's own name | -0.085 | 0.207 | Yes |
| Riva et al., 2018 | 2018 | 42 | 1.03 | auditory | frequency, duration | tone | 0.292 | 0.075 | No |
| Grisoni et al., 2019 | 2019 | 42 | 34.95 | auditory | semantic | speech | -0.414 | 0.165 | No |
| Matsuzaki, Ku, et al., 2019 | 2019 | 25 | 24.74 | auditory | vowel | speech | -0.386 | 0.243 | No |
| Matsuzaki, Kuschner, et al., 2019 | 2019 | 46 | 10.22 | auditory | vowel | speech | -0.196 | 0.218 | No |
| Zhang et al., 2019 | 2019 | 32 | 9.95 | auditory | vowel, frequency | speech, tone | 0.16 | 0.042 | No |
| Di Lorenzo et al., 2020* | 2020 | 38 | 14.17 | auditory | duration, frequency | tone | -0.407 | 0.035 | No |
| Green et al., 2020* | 2020 | 17 | 7.92 | auditory | vowel | speech | 0.422 | 0.098 | No |
| Knight et al., 2020* | 2020 | 40 | 14.5 | auditory | frequency | tone | -0.566 | 0.087 | No |
| Ruiz-Martínez et al., 2020 | 2020 | 31 | 8.91 | auditory | frequency | tone | -0.087 | 0.056 | No |
| Cary et al., n.d. | 2021 | 26 | 12.67 | auditory | frequency | tone | -0.519 | 0.166 | No |
| Kadlaskar et al., 2021* | 2021 | 28 | 10.04 | auditory | vowel | speech | 13.93 | 4.542 | No |
| Piatti et al., 2021 | 2021 | 34 | 3.13 | auditory | frequency, vowel | tone, speech | -0.347 | 0.11 | No |
| Goris et al., 2022 | 2022 | 54 | 35.63 | auditory | frequency | tone | -0.183 | 0.144 | No |
| Haigh et al., 2022 | 2022 | 52 | 28.6 | auditory | frequency, vowel | tone, speech | -0.355 | 0.069 | No |
| Lassen et al., 2022* | 2022 | 118 | 11.82 | auditory | frequency | tone | -1.03 | 0.108 | No |
| Haigh et al., 2022 | 2023 | 52 | 31.55 | auditory | frequency | tone | 0 | 0 | No |
| Irwin et al., 2021^*^ | 2023 | 47 | 9.55 | auditory | vowel | speech | 1.193 | 0.091 | Yes |
| Kabil et al., 2023^*^ | 2023 | 120 | 9.92 | auditory | frequency | tone | -0.64 | 0.162 | No |
| Mayerle, 2023^*^ | 2023 | 68 | 11.06 | auditory | frequency | tone | 0.234 | 0.034 | No |
| Cary et al., 2024 | 2024 | 26 | 12.67 | auditory | frequency | tone | 0.513 |  | No |
| Sokhadze et al., 2009^*^ | 2009 | 22 | 18.1 | visual | shape | letter | -1.862 | 0.187 | Yes |
| Baruth et al., 2010 | 2010 | 30 | 14.7 | visual | orientation | shape | 0 | 0 | Yes |
| E. Sokhadze et al., 2010^*^ | 2010 | 28 | 13.55 | visual | shape | letter | -1.565 | 0.289 | Yes |
| Key & Stone, 2012^*^ | 2012 | 35 | 0.75 | visual | identity | face | -0.226 | 0.126 | Yes |
| Clery, Andersson, et al., 2013 | 2013 | 24 | 11.42 | visual | shape | shape | 0.52 | 0.088 | Yes |
| Clery, Roux, et al., 2013^*^ | 2013 | 26 | 25.21 | visual | shape | shape | 1.288 | 0.256 | Yes |
| Westerfield et al., 2015^*^ | 2015 | 32 | 32.55 | visual | shape | shape | 0.926 | 0.161 | Yes |
| E. M. Sokhadze et al., 2017 | 2017 | 56 | 13.5 | visual | shape | letter | 0.038 | 0.157 | Yes |
| S. Wang et al., 2017 | 2017 | 32 | 5.45 | visual | color | cartoon | -0.224 | 0.119 | No |
| Dwyer et al., 2019^*^ | 2019 | 32 | 29.56 | visual | orientation | face | -1.003 | 0.099 | No |
| Van der Donck et al., 2019 | 2019 | 46 | 10.5 | visual | emotion | face | 0.545 | 0.199 | No |
| Kovarski et al., 2021 | 2021 | 34 | 26 | visual | emotion | face | -0.018 | 0.167 | No |
| Hecker et al., 2022 | 2022 | 35 | 40.05 | visual | frequency | checkerboard | 0.043 | 0.125 | No |
| * Studies denoted with asterisk indicate potential outliers as identified by model | | | | | | | | | |

*Supplementary Table 2 References*

Abdeltawwab, M. M., & Baz, H. (2015). Automatic pre-attentive auditory responses: MMN to tone burst frequency changes in autistic school-age children. *J Int Adv Otol*, *11*(1), 36–41.

Baruth, J., Casanova, M., Sears, L., & Sokhadze, E. (2010). *Early-stage visual processing abnormalities in high-functioning autism spectrum disorder (ASD)*. *1*(2), 177–187. https://doi.org/10.2478/v10134-010-0024-9

Cary, E., Pacheco, D., Kaplan-Kahn, E., McKernan, E., Matsuba, E., Prieve, B., & Russo, N. (2024). Brain Signatures of Early and Late Neural Measures of Auditory Habituation and Discrimination in Autism and Their Relationship to Autistic Traits and Sensory Overresponsivity. *Journal of Autism and Developmental Disorders*, *54*(4), 1344–1360. https://doi.org/10.1007/s10803-022-05866-8

Cary, E., Pacheco, D., Kaplan-Kahn, E., McKernan, E., Prieve, B., & Russo, N. (n.d.). Timing is everything: Early and late neural measures of auditory habituation and discrimination in autism and their relationship to autistic traits and sensory overresponsivity. *Preprint*. https://doi.org/10.21203/rs.3.rs-547935/v1

Čeponienė, R., Lepistö, T., Shestakova, A., Vanhala, R., Alku, P., Näätänen, R., & Yaguchi, K. (2003). Speech–sound-selective auditory impairment in children with autism: They can perceive but do not attend. *Proceedings of the National Academy of Sciences*, *100*(9), 5567–5572. https://doi.org/10.1073/pnas.0835631100

Charpentier, J., Kovarski, K., Houy-Durand, E., Malvy, J., Saby, A., Bonnet-Brilhault, F., Latinus, M., & Gomot, M. (2018). Emotional prosodic change detection in autism Spectrum disorder: An electrophysiological investigation in children and adults. *Journal of Neurodevelopmental Disorders*, *10*(1), 28. https://doi.org/10.1186/s11689-018-9246-9

Clery, H., Andersson, F., Bonnet-Brilhault, F., Philippe, A., Wicker, B., & Gomot, M. (2013). fMRI investigation of visual change detection in adults with autism. *NeuroImage: Clinical*, *2*, 303–312. https://doi.org/10.1016/j.nicl.2013.01.010

Clery, H., Roux, S., Houy-Durand, E., Bonnet-Brilhault, F., Bruneau, N., & Gomot, M. (2013). Electrophysiological evidence of atypical visual change detection in adults with autism. *Frontiers in Human Neuroscience*, *7*. https://www.frontiersin.org/articles/10.3389/fnhum.2013.00062

Courchesne, E., Kilman, B. A., Galambos, R., & Lincoln, A. J. (1984). Autism: Processing of novel auditory information assessed by event-related brain potentials. *Electroencephalography and Clinical Neurophysiology/Evoked Potentials Section*, *59*(3), 238–248. https://doi.org/10.1016/0168-5597(84)90063-7

Di Lorenzo, G., Riccioni, A., Ribolsi, M., Siracusano, M., Curatolo, P., & Mazzone, L. (2020). Auditory Mismatch Negativity in Youth Affected by Autism Spectrum Disorder With and Without Attenuated Psychosis Syndrome. *Frontiers in Psychiatry*, *11*. https://www.frontiersin.org/articles/10.3389/fpsyt.2020.555340

Donkers, F. C. L., Schipul, S. E., Baranek, G. T., Cleary, K. M., Willoughby, M. T., Evans, A. M., Bulluck, J. C., Lovmo, J. E., & Belger, A. (2015). Attenuated Auditory Event-Related Potentials and Associations with Atypical Sensory Response Patterns in Children with Autism. *Journal of Autism and Developmental Disorders*, *45*(2), 506–523. https://doi.org/10.1007/s10803-013-1948-y

Dunn, M. A., Gomes, H., & Gravel, J. (2008). Mismatch Negativity in Children with Autism and Typical Development. *Journal of Autism and Developmental Disorders*, *38*(1), 52–71. https://doi.org/10.1007/s10803-007-0359-3

Dwyer, P., Xu, B., & Tanaka, J. W. (2019). Investigating the perception of face identity in adults on the autism spectrum using behavioural and electrophysiological measures. *Face Perception: Experience, Models and Neural Mechanisms*, *157*, 132–141. https://doi.org/10.1016/j.visres.2018.02.013

Erwin, R., Van Lancker, D., Guthrie, D., Schwafel, J., Tanguay, P., & Buchwald, J. S. (1991). P3 responses to prosodic stimuli in adult autistic subjects. *Electroencephalography and Clinical Neurophysiology/Evoked Potentials Section*, *80*(6), 561–571. https://doi.org/10.1016/0168-5597(91)90139-O

Fan, Y.-T., & Cheng, Y. (2014). Atypical Mismatch Negativity in Response to Emotional Voices in People with Autism Spectrum Conditions. *PLOS ONE*, *9*(7), e102471. https://doi.org/10.1371/journal.pone.0102471

Ferri, R., Elia, M., Agarwal, N., Lanuzza, B., Musumeci, S. A., & Pennisi, G. (2003). The mismatch negativity and the P3a components of the auditory event-related potentials in autistic low-functioning subjects. *Clinical Neurophysiology*, *114*(9), 1671–1680. https://doi.org/10.1016/S1388-2457(03)00153-6

Gomot, M., Belmonte, M. K., Bullmore, E. T., Bernard, F. A., & Baron-Cohen, S. (2008). Brain hyper-reactivity to auditory novel targets in children with high-functioning autism. *Brain*, *131*(9), 2479–2488. https://doi.org/10.1093/brain/awn172

Gomot, M., Bernard, F. A., Davis, M. H., Belmonte, M. K., Ashwin, C., Bullmore, E. T., & Baron-Cohen, S. (2006). Change detection in children with autism: An auditory event-related fMRI study. *NeuroImage*, *29*(2), 475–484. https://doi.org/10.1016/j.neuroimage.2005.07.027

Gomot, M., Blanc, R., Clery, H., Roux, S., Barthelemy, C., & Bruneau, N. (2011). Candidate Electrophysiological Endophenotypes of Hyper-Reactivity to Change in Autism. *Journal of Autism and Developmental Disorders*, *41*(6), 705–714. https://doi.org/10.1007/s10803-010-1091-y

Gomot, M., Giard, M.-H., Adrien, J.-L., Barthelemy, C., & Bruneau, N. (2002). Hypersensitivity to acoustic change in children with autism: Electrophysiological evidence of left frontal cortex dysfunctioning. *Psychophysiology*, *39*(5), 577–584. https://doi.org/10.1111/1469-8986.3950577

Gonzalez-Gadea, M. L., Chennu, S., Bekinschtein, T. A., Rattazzi, A., Beraudi, A., Tripicchio, P., Moyano, B., Soffita, Y., Steinberg, L., Adolfi, F., Sigman, M., Marino, J., Manes, F., & Ibanez, A. (2015). Predictive coding in autism spectrum disorder and attention deficit hyperactivity disorder. *Journal of Neurophysiology*, *114*(5), 2625–2636. https://doi.org/10.1152/jn.00543.2015

Goris, J., Braem, S., Nijhof, A. D., Rigoni, D., Deschrijver, E., Van de Cruys, S., Wiersema, J. R., & Brass, M. (2018). Sensory Prediction Errors Are Less Modulated by Global Context in Autism Spectrum Disorder. *Biological Psychiatry: Cognitive Neuroscience and Neuroimaging*, *3*(8), 667–674. https://doi.org/10.1016/j.bpsc.2018.02.003

Goris, J., Braem, S., Van Herck, S., Simoens, J., Deschrijver, E., Wiersema, J. R., Paton, B., Brass, M., & Todd, J. (2022). Reduced Primacy Bias in Autism during Early Sensory Processing. *The Journal of Neuroscience*, *42*(19), 3989. https://doi.org/10.1523/JNEUROSCI.3088-20.2022

Green, H. L., Shuffrey, L. C., Levinson, L., Shen, G., Avery, T., Randazzo Wagner, M., Sepulveda, D. M., Garcia, P., Maddox, C., Garcia, F., Hassan, S., & Froud, K. (2020). Evaluation of mismatch negativity as a marker for language impairment in autism spectrum disorder. *Journal of Communication Disorders*, *87*, 105997. https://doi.org/10.1016/j.jcomdis.2020.105997

Grisoni, L., Moseley, R. L., Motlagh, S., Kandia, D., Sener, N., Pulvermüller, F., Roepke, S., & Mohr, B. (2019). Prediction and Mismatch Negativity Responses Reflect Impairments in Action Semantic Processing in Adults With Autism Spectrum Disorders. *Frontiers in Human Neuroscience*, *13*. https://www.frontiersin.org/articles/10.3389/fnhum.2019.00395

Guiraud, J. A., Kushnerenko, E., Tomalski, P., Davies, K., Ribeiro, H., Johnson, M. H., & The BASIS Team. (2011). Differential habituation to repeated sounds in infants at high risk for autism. *NeuroReport*, *22*(16). https://journals.lww.com/neuroreport/Fulltext/2011/11160/Differential_habituation_to_repeated_sounds_in.12.aspx

Haigh, S. M., Brosseau, P., Eack, S. M., Leitman, D. I., Salisbury, D. F., & Behrmann, M. (2022). Hyper-Sensitivity to Pitch and Poorer Prosody Processing in Adults With Autism: An ERP Study. *Frontiers in Psychiatry*, *13*. https://www.frontiersin.org/articles/10.3389/fpsyt.2022.844830

Hecker, L., Wilson, M., Tebartz van Elst, L., & Kornmeier, J. (2022). Altered EEG variability on different time scales in participants with autism spectrum disorder: An exploratory study. *Scientific Reports*, *12*(1), 13068. https://doi.org/10.1038/s41598-022-17304-x

Huang, D., Yu, L., Wang, X., Fan, Y., Wang, S., & Zhang, Y. (2018). Distinct patterns of discrimination and orienting for temporal processing of speech and nonspeech in Chinese children with autism: An event‐related potential study. *European Journal of Neuroscience*, *47*(6), 662–668.

Hudac, C. M., DesChamps, T. D., Arnett, A. B., Cairney, B. E., Ma, R., Webb, S. J., & Bernier, R. A. (2018). Early enhanced processing and delayed habituation to deviance sounds in autism spectrum disorder. *Brain and Cognition*, *123*, 110–119.

Irwin, J., Avery, T., Kleinman, D., & Landi, N. (2021). Audiovisual Speech Perception in Children with Autism Spectrum Disorders: Evidence from Visual Phonemic Restoration. *Journal of Autism and Developmental Disorders*. https://doi.org/10.1007/s10803-021-04916-x

Iwanami, A., Okajima, Y., Ota, H., Tani, M., Yamada, T., Yamagata, B., Hashimoto, R., Kanai, C., Takashio, O., Inamoto, A., Ono, T., Takayama, Y., & Kato, N. (2014). P300 Component of Event-Related Potentials in Persons With Asperger Disorder. *Journal of Clinical Neurophysiology*, *31*(5). https://journals.lww.com/clinicalneurophys/fulltext/2014/10000/p300_component_of_event_related_potentials_in.17.aspx

Jansson-Verkasalo, E., Ceponiene, R., Kielinen, M., Suominen, K., Jäntti, V., Linna, S.-L., Moilanen, I., & Näätänen, R. (2003). Deficient auditory processing in children with Asperger Syndrome, as indexed by event-related potentials. *Neuroscience Letters*, *338*(3), 197–200. https://doi.org/10.1016/S0304-3940(02)01405-2

Jansson-Verkasalo, E., Kujala, T., Jussila, K., Mattila, M. L., Moilanen, I., Näätänen, R., Suominen, K., & Korpilahti, P. (2005). Similarities in the phenotype of the auditory neural substrate in children with Asperger syndrome and their parents. *European Journal of Neuroscience*, *22*(4), 986–990. https://doi.org/10.1111/j.1460-9568.2005.04216.x

Kabil, S., Abdelshafy, R., Ahmed, A., Zahran, A., Attalah, M., Sallam, Y., & El Lateef, A. (2023). Mismatch Negativity and Auditory Brain Stem Response in Children with Autism Spectrum Disorders and Language Disorders. *J Multidiscip Healthc.*, *16*, 811–817. https://doi.org/10.2147/JMDH.S401937

Kadlaskar, G., Bergmann, S., McNally Keehn, R., Seidl, A., & Keehn, B. (2021). Electrophysiological Measures of Tactile and Auditory Processing in Children With Autism Spectrum Disorder. *Frontiers in Human Neuroscience*, *15*. https://www.frontiersin.org/articles/10.3389/fnhum.2021.729270

Kasai, K., Hashimoto, O., Kawakubo, Y., Yumoto, M., Kamio, S., Itoh, K., Koshida, I., Iwanami, A., Nakagome, K., & Fukuda, M. (2005). Delayed automatic detection of change in speech sounds in adults with autism: A magnetoencephalographic study. *Clinical Neurophysiology*, *116*(7), 1655–1664.

Key, A. P. F., & Stone, W. L. (2012). Processing of novel and familiar faces in infants at average and high risk for autism. *Developmental Cognitive Neuroscience*, *2*(2), 244–255. https://doi.org/10.1016/j.dcn.2011.12.003

Knight, E. J., Oakes, L., Hyman, S. L., Freedman, E. G., & Foxe, J. J. (2020). Individuals With Autism Have No Detectable Deficit in Neural Markers of Prediction Error When Presented With Auditory Rhythms of Varied Temporal Complexity. *Autism Research*, *13*(12), 2058–2072. https://doi.org/10.1002/aur.2362

Korpilahti, P., Jansson-Verkasalo, E., Mattila, M.-L., Kuusikko, S., Suominen, K., Rytky, S., Pauls, D. L., & Moilanen, I. (2007). Processing of Affective Speech Prosody is Impaired in Asperger Syndrome. *Journal of Autism and Developmental Disorders*, *37*(8), 1539–1549. https://doi.org/10.1007/s10803-006-0271-2

Kovarski, K., Charpentier, J., Roux, S., Batty, M., Houy-Durand, E., & Gomot, M. (2021). Emotional visual mismatch negativity: A joint investigation of social and non-social dimensions in adults with autism. *Translational Psychiatry*, *11*(1), 10. https://doi.org/10.1038/s41398-020-01133-5

Kuhl, P. K., Coffey-Corina, S., Padden, D., & Dawson, G. (2005). Links between social and linguistic processing of speech in preschool children with autism: Behavioral and electrophysiological measures. *Developmental Science*, *8*(1), F1–F12. https://doi.org/10.1111/j.1467-7687.2004.00384.x

Kujala, T., Aho, E., Lepistö, T., Jansson-Verkasalo, E., Nieminen-von Wendt, T., von Wendt, L., & Näätänen, R. (2007). Atypical pattern of discriminating sound features in adults with Asperger syndrome as reflected by the mismatch negativity. *Biological Psychology*, *75*(1), 109–114. https://doi.org/10.1016/j.biopsycho.2006.12.007

Kujala, T., Kuuluvainen, S., Saalasti, S., Jansson-Verkasalo, E., Wendt, L. von, & Lepistö, T. (2010). Speech-feature discrimination in children with Asperger syndrome as determined with the multi-feature mismatch negativity paradigm. *Clinical Neurophysiology*, *121*(9), 1410–1419. https://doi.org/10.1016/j.clinph.2010.03.017

Kujala, T., Lepistö, T., Nieminen-von Wendt, T., Näätänen, P., & Näätänen, R. (2005). Neurophysiological evidence for cortical discrimination impairment of prosody in Asperger syndrome. *Neuroscience Letters*, *383*(3), 260–265. https://doi.org/10.1016/j.neulet.2005.04.048

Lassen, J., Oranje, B., Vestergaard, M., Foldager, M., Kjær, T. W., Arnfred, S., & Aggernæs, B. (2022). Reduced mismatch negativity in children and adolescents with autism spectrum disorder is associated with their impaired adaptive functioning. *Autism Research*, *15*(8), 1469–1481. https://doi.org/10.1002/aur.2738

Lepistö, T., Kajander, M., Vanhala, R., Alku, P., Huotilainen, M., Näätänen, R., & Kujala, T. (2008). The perception of invariant speech features in children with autism. *Biological Psychology*, *77*(1), 25–31. https://doi.org/10.1016/j.biopsycho.2007.08.010

Lepistö, T., Kujala, T., Vanhala, R., Alku, P., Huotilainen, M., & Näätänen, R. (2005). The discrimination of and orienting to speech and non-speech sounds in children with autism. *Brain Research*, *1066*(1), 147–157. https://doi.org/10.1016/j.brainres.2005.10.052

Lepistö, T., Nieminen-von Wendt, T., von Wendt, L., Näätänen, R., & Kujala, T. (2007). Auditory cortical change detection in adults with Asperger syndrome. *Neuroscience Letters*, *414*(2), 136–140. https://doi.org/10.1016/j.neulet.2006.12.009

Lepistö, T., Silokallio, S., Nieminen-von Wendt, T., Alku, P., Näätänen, R., & Kujala, T. (2006). Auditory perception and attention as reflected by the brain event-related potentials in children with Asperger syndrome. *Clinical Neurophysiology*, *117*(10), 2161–2171. https://doi.org/10.1016/j.clinph.2006.06.709

Lindström, R., Lepistö-Paisley, T., Makkonen, T., Reinvall, O., Nieminen-von Wendt, T., Alén, R., & Kujala, T. (2018). Atypical perceptual and neural processing of emotional prosodic changes in children with autism spectrum disorders. *Clinical Neurophysiology*, *129*(11), 2411–2420. https://doi.org/10.1016/j.clinph.2018.08.018

Lindström, R., Lepistö-Paisley, T., Vanhala, R., Alén, R., & Kujala, T. (2016). Impaired neural discrimination of emotional speech prosody in children with autism spectrum disorder and language impairment. *Neuroscience Letters*, *628*, 47–51. https://doi.org/10.1016/j.neulet.2016.06.016

Ludlow, A., Mohr, B., Whitmore, A., Garagnani, M., Pulvermüller, F., & Gutierrez, R. (2014). Auditory processing and sensory behaviours in children with autism spectrum disorders as revealed by mismatch negativity. *Brain and Cognition*, *86*, 55–63. https://doi.org/10.1016/j.bandc.2014.01.016

Matsuzaki, J., Kagitani-Shimono, K., Sugata, H., Hanaie, R., Nagatani, F., Yamamoto, T., Tachibana, M., Tominaga, K., Hirata, M., Mohri, I., & Taniike, M. (2017). Delayed Mismatch Field Latencies in Autism Spectrum Disorder with Abnormal Auditory Sensitivity: A Magnetoencephalographic Study. *Frontiers in Human Neuroscience*, *11*. https://www.frontiersin.org/articles/10.3389/fnhum.2017.00446

Matsuzaki, J., Ku, M., Berman, J. I., Blaskey, L., Bloy, L., Chen, Y., Dell, J., Edgar, J. C., Kuschner, E. S., Liu, S., Saby, J., Brodkin, E. S., & Roberts, T. P. L. (2019). Abnormal auditory mismatch fields in adults with autism spectrum disorder. *Neuroscience Letters*, *698*, 140–145. https://doi.org/10.1016/j.neulet.2018.12.043

Matsuzaki, J., Kuschner, E. S., Blaskey, L., Bloy, L., Kim, M., Ku, M., Edgar, J. C., Embick, D., & Roberts, T. P. L. (2019). Abnormal auditory mismatch fields are associated with communication impairment in both verbal and minimally verbal/nonverbal children who have autism spectrum disorder. *Autism Research*, *12*(8), 1225–1235. https://doi.org/10.1002/aur.2136

Mayerle, M. C. C. da S. R., Rudimar; Gregory, Letícia; Borges, Viviann Magalhães Silva; Sleifer, Pricila. (2023). Mismatch Negativity in Children and Adolescents with Autism Spectrum Disorder. *International Archives of Otorhinolaryngology*, *27*(02), e218–e225. https://doi.org/10.1055/s-0043-1768209

Nijhof, A. D., Dhar, M., Goris, J., Brass, M., & Wiersema, J. R. (2018). Atypical neural responding to hearing one’s own name in adults with ASD. *Journal of Abnormal Psychology*, *127*(1), 129.

Oram Cardy, J. E., Flagg, E. J., Roberts, W., & Roberts, T. P. L. (2005). Delayed mismatch field for speech and non-speech sounds in children with autism. *NeuroReport*, *16*(5). https://journals.lww.com/neuroreport/Fulltext/2005/04040/Delayed_mismatch_field_for_speech_and_non_speech.21.aspx

Piatti, A., Van der Paelt, S., Warreyn, P., & Roeyers, H. (2021). Atypical attention to voice in toddlers and pre-schoolers with autism spectrum disorder is related to unimpaired cognitive abilities. An ERP study. *Research in Autism Spectrum Disorders*, *86*, 101805. https://doi.org/10.1016/j.rasd.2021.101805

Riva, V., Cantiani, C., Mornati, G., Gallo, M., Villa, L., Mani, E., Saviozzi, I., Marino, C., & Molteni, M. (2018). Distinct ERP profiles for auditory processing in infants at-risk for autism and language impairment. *Scientific Reports*, *8*(1), 715. https://doi.org/10.1038/s41598-017-19009-y

Roberts, T. P. L., Cannon, K. M., Tavabi, K., Blaskey, L., Khan, S. Y., Monroe, J. F., Qasmieh, S., Levy, S. E., & Edgar, J. C. (2011). Auditory Magnetic Mismatch Field Latency: A Biomarker for Language Impairment in Autism. *Genotype, Circuits, and Cognition in Autism and Attention-Deficit/Hyperactivity Disorder*, *70*(3), 263–269. https://doi.org/10.1016/j.biopsych.2011.01.015

Ruiz-Martínez, F. J., Rodríguez-Martínez, E. I., Wilson, C. E., Yau, S., Saldaña, D., & Gómez, C. M. (2020). Impaired P1 Habituation and Mismatch Negativity in Children with Autism Spectrum Disorder. *Journal of Autism and Developmental Disorders*, *50*(2), 603–616. https://doi.org/10.1007/s10803-019-04299-0

Sokhadze, E., Baruth, J., El-Baz, A., Horrell, T., Sokhadze, G., Carroll, T., Tasman, A., Sears, L., & Casanova, M. F. (2010). Impaired error monitoring and correction function in autism. *Journal of Neurotherapy*, *14*(2), 79–95.

Sokhadze, E., Baruth, J., Tasman, A., Sears, L., Mathai, G., El-Baz, A., & Casanova, M. F. (2009). Event-related Potential Study of Novelty Processing Abnormalities in Autism. *Applied Psychophysiology and Biofeedback*, *34*(1), 37–51. https://doi.org/10.1007/s10484-009-9074-5

Sokhadze, E. M., Lamina, E. V., Casanova, E. L., Kelly, D. P., Opris, I., Khachidze, I., & Casanova, M. F. (2017). Atypical Processing of Novel Distracters in a Visual Oddball Task in Autism Spectrum Disorder. *Behavioral Sciences*, *7*(4). https://doi.org/10.3390/bs7040079

Van der Donck, S., Dzhelyova, M., Vettori, S., Thielen, H., Steyaert, J., Rossion, B., & Boets, B. (2019). Fast Periodic Visual Stimulation EEG Reveals Reduced Neural Sensitivity to Fearful Faces in Children with Autism. *Journal of Autism and Developmental Disorders*, *49*(11), 4658–4673. https://doi.org/10.1007/s10803-019-04172-0

Vlaskamp, C., Oranje, B., Madsen, G. F., Møllegaard Jepsen, J. R., Durston, S., Cantio, C., Glenthøj, B., & Bilenberg, N. (2017). Auditory processing in autism spectrum disorder: Mismatch negativity deficits. *Autism Research*, *10*(11), 1857–1865. https://doi.org/10.1002/aur.1821

Wang, S., Yang, C., Liu, Y., Shao, Z., & Jackson, T. (2017). Early and late stage processing abnormalities in autism spectrum disorders: An ERP study. *PLOS ONE*, *12*(5), e0178542. https://doi.org/10.1371/journal.pone.0178542

Wang, X., Wang, S., Fan, Y., Huang, D., & Zhang, Y. (2017). Speech-specific categorical perception deficit in autism: An Event-Related Potential study of lexical tone processing in Mandarin-speaking children. *Scientific Reports*, *7*(1), 43254. https://doi.org/10.1038/srep43254

Westerfield, M. A., Zinni, M., Vo, K., & Townsend, J. (2015). Tracking the Sensory Environment: An ERP Study of Probability and Context Updating in ASD. *Journal of Autism and Developmental Disorders*, *45*(2), 600–611. https://doi.org/10.1007/s10803-014-2045-6

Whitehouse, A. J., Line, E., Watt, H. J., & Bishop, D. V. (2009). Qualitative aspects of developmental language impairment relate to language and literacy outcome in adulthood. *International Journal of Language & Communication Disorders*, *44*(4), 489–510.

Yoshimura, Y., Kikuchi, M., Hayashi, N., Hiraishi, H., Hasegawa, C., Takahashi, T., Oi, M., Remijn, G. B., Ikeda, T., Saito, D. N., Kumazaki, H., & Minabe, Y. (2017). Altered human voice processing in the frontal cortex and a developmental language delay in 3- to 5-year-old children with autism spectrum disorder. *Scientific Reports*, *7*(1), 17116. https://doi.org/10.1038/s41598-017-17058-x

Zhang, J., Meng, Y., Wu, C., Xiang, Y.-T., & Yuan, Z. (2019). Non-speech and speech pitch perception among Cantonese-speaking children with autism spectrum disorder: An ERP study. *Neuroscience Letters*, *703*, 205–212. https://doi.org/10.1016/j.neulet.2019.03.021
